# Classification of Individuals With COVID-19 and Post–COVID-19 Condition and Healthy Controls Using Heart Rate Variability: Machine Learning Study With a Near–Real-Time Monitoring Component

**DOI:** 10.2196/76613

**Published:** 2025-08-14

**Authors:** Carlos Alberto Sanches, Andre Felipe Henriques Librantz, Luciana Maria Malosá Sampaio, Peterson Adriano Belan

**Affiliations:** 1 Informatics and Knowledge Management Graduate Program Universidade Nove de Julho São Paulo Brazil; 2 Postgraduate Program in Rehabilitation Sciences Universidade Nove de Julho São Paulo Brazil

**Keywords:** heart rate variability, physiological signature, COVID-19 sequelae, decision tree model, digital health monitoring

## Abstract

**Background:**

Heart rate variability (HRV) is a validated biomarker of autonomic and inflammatory regulation and has been associated with both acute COVID-19 and post–COVID-19 condition. Although reverse transcription polymerase chain reaction remains the diagnostic gold standard for acute infection, there is a lack of accessible, noninvasive physiological tools to support ongoing monitoring and stage differentiation of COVID-19 and its sequelae. The growing availability of wearable devices capable of real-time HRV data collection opens up opportunities for early detection and health status classification using machine learning.

**Objective:**

This study aimed to identify HRV patterns capable of distinguishing individuals with active COVID-19 and post–COVID-19 condition and healthy controls using data collected from wearable devices and processed using machine learning models. A secondary objective was to assess the feasibility of a near–real-time health monitoring system based on these patterns using wearable-derived HRV data.

**Methods:**

HRV indexes (SD of the normal-to-normal intervals [SDNN], root mean square of successive differences [RMSSD], low-frequency relative power [LF%], and high-frequency relative power [HF%]) were collected from 61 participants (n=21, 34% with active COVID-19; n=20, 33% with post–COVID-19 condition; and n=20, 33% healthy controls) using 2 standardized datasets. Classification models were developed using supervised machine learning algorithms (decision tree, support vector machines, k-nearest neighbor, and neural networks) and evaluated through cross-validation. A contextual clinical variable indicating recent SARS-CoV-2 infection was incorporated into 1 model configuration to assess its impact on classification performance. In addition, a prototype system for near–real-time monitoring was implemented and tested in a separate group of 4 participants.

**Results:**

Participants with active COVID-19 showed significantly lower HRV indexes (SDNN, RMSSD, LF%, and HF%) compared to both participants with post–COVID-19 condition and healthy controls (*P*<.001), whereas differences between the post–COVID-19 condition and healthy groups were not statistically significant. Decision tree models trained solely on HRV features achieved 76.4% accuracy with high discriminative performance for active COVID-19 (*F*_1_-score=88%; area under the curve=0.85) but limited detection of post–COVID-19 condition (*F*_1_-score=56%). When a contextual clinical variable indicating recent SARS-CoV-2 infection was included, overall accuracy increased to 87%, and the *F*_1_-score for post–COVID-19 condition rose to 92%, with improved area under the curve metrics across classes. A prototype system tested on 4 independent participants correctly classified their status, demonstrating feasibility for near–real-time application.

**Conclusions:**

HRV patterns collected from wearable devices and analyzed via machine learning successfully distinguished individuals with active COVID-19 from healthy individuals with high accuracy using physiological data alone. When a clinical contextual variable indicating recent infection was added, the model also achieved strong performance in identifying post–COVID-19 condition cases. A prototype system demonstrated feasibility for near–real-time application, reinforcing the potential of HRV for individualized health monitoring.

## Introduction

### Background

The COVID-19 pandemic continues to pose a significant global challenge, with the World Health Organization reporting >3.5 million new cases and nearly 69,000 deaths in 2024 alone [1,2]. Although multiple testing strategies are available for detecting acute infection, such as reverse transcription polymerase chain reaction (RT-PCR), antigen tests, and serological assays, each presents limitations in cost, sensitivity, availability, or applicability at different stages of the disease [3-5]. Mass availability of these tests is not straightforward as it involves complex production, logistics, and cost-related challenges. Furthermore, these tests are not useful for diagnosing post–COVID-19 condition.

Even after recovering from acute COVID-19, a significant proportion of patients continue to experience physical, psychological, and cognitive symptoms, which is known as post–COVID-19 condition [6,7]. This syndrome is characterized by persistent manifestations beyond microbiological recovery [8]. According to the World Health Organization, post–COVID-19 condition occurs in individuals with a confirmed history of SARS-CoV-2 infection, typically 3 months after onset, with symptoms lasting at least 2 months and not explained by alternative diagnoses [9].

Estimates suggest that at least 65 million people worldwide have post–COVID-19 condition, with the number increasing daily, whereas current diagnostic and treatment options remain insufficient [10]. Meta-analyses have shown that a substantial proportion of survivors of COVID-19 experience residual symptoms that persist for weeks, months, or even a year after recovery from the disease [11-13]. A systematic review [14] showed that post–COVID-19 condition can develop even after mild or asymptomatic SARS-CoV-2 infection.

However, no precise or reliable diagnostic test exists for post–COVID-19 condition, and the assessment of its prevalence remains controversial. Differences in methodologies and case definitions have led to wide variations in reported prevalence, ranging from <10% to approximately 60% of COVID-19 cases [15].

Given these challenges, wearable devices have emerged as a promising tool for continuous health monitoring. The adoption of personal devices that collect physiological data has been increasing annually [16]. These devices enable the noninvasive collection of real-time data that, when appropriately processed, can be used to assess physiological changes, including overall health status [17].

Several studies have investigated the use of physiological data to detect COVID-19, with some reporting successful prediction of infection even before symptom onset [18-24]. These studies analyzed various physiological parameters, including skin temperature, respiratory rate, and heart rate. Among the various biomarkers, heart rate variability (HRV) has gained attention for its potential as an indicator of autonomic nervous system (ANS) activity [25-27].

HRV is a noninvasive, objective, and validated measure of ANS dysfunction, providing insights into the balance between the sympathetic and parasympathetic nervous systems through the variation in time intervals between consecutive heartbeats [28]. While high HRV is associated with good health, a reduction in HRV is linked to various health issues [29,30].

The relationship between HRV and inflammatory markers has been extensively studied. A meta-analysis of 51 studies demonstrated an inverse correlation between HRV indexes and inflammatory markers, including interleukin-6 and C-reactive protein (CRP) [31]. Similarly, other studies have indicated HRV as a sensitive biomarker of inflammatory activity and immunomodulation, with variations in certain HRV parameters associated with elevated levels of proinflammatory cytokines, such as interleukin-6 and CRP [32-34]. Furthermore, there is evidence suggesting that lower HRV is correlated with higher CRP levels, reinforcing the connection between autonomic regulation and inflammatory control [35].

These findings highlight the importance of HRV as a physiological marker of the inflammatory response, with potential applications in monitoring inflammatory and infectious conditions. In addition, HRV reduction has been observed to precede clinical deterioration, suggesting its potential as an early physiological marker of disease progression [36].

Recent evidence indicates that COVID-19 significantly impacts HRV, reflecting autonomic and inflammatory dysregulation during the course of infection. A study of 143 individuals, including 93 patients with COVID-19 and 50 healthy controls, demonstrated that SARS-CoV-2 infection is associated with HRV alterations, indicating potential autonomic and inflammatory impairment [37].

These findings are consistent with a growing body of evidence linking SARS-CoV-2 infection to impaired autonomic function and reduced HRV [38-42]. Moreover, some studies report that HRV changes can precede symptom onset, reinforcing its value for early detection [43,44]. A recent systematic review and meta-analysis further supports the feasibility of using wearable-derived HRV data to detect and monitor COVID-19 [45].

Beyond its utility as a biomarker of SARS-CoV-2 infection, there is evidence suggesting that persistent HRV alterations following SARS-CoV-2 infection may serve as a valuable marker for what is commonly known as post–COVID-19 condition. Several studies analyzing HRV in patients clinically diagnosed with post–COVID-19 condition have consistently shown reduced HRV compared to healthy controls [46-52].

These findings suggest enduring autonomic dysfunction and inflammatory dysregulation in individuals with this condition, indicating that HRV may serve as a marker for identifying and monitoring post–COVID-19 condition. However, most studies identifying these conditions have relied on statistical methods to associate HRV variations with the disease. Furthermore, most existing research has focused on retrospective analyses where physiological data were collected; processed; and subsequently compared to self-reported symptoms, laboratory-confirmed COVID-19 cases, or clinical diagnoses of post–COVID-19 condition.

Building on these efforts, this study focused on identifying distinct HRV patterns among healthy individuals, patients with COVID-19, and those with post–COVID-19 condition. These patterns may reflect autonomic and inflammatory regulation, providing the basis for a proposed machine learning–based predictive model for COVID-19 and post–COVID-19 condition.

The primary contribution of this study lies in delineating these HRV patterns, which capture meaningful physiological changes and enable predictive capabilities. By leveraging wearable devices, this approach facilitates noninvasive health monitoring, representing a significant advancement in the early detection and long-term management of COVID-19 and post–COVID-19 condition with near–real-time prediction capabilities. Unlike traditional diagnostic approaches, this method offers a pioneering advantage by providing, for the first time, predictions of post–COVID-19 condition based solely on physiological data rather than relying exclusively on clinical assessments.

### Prior Work

#### Overview

HRV has emerged as a valuable biomarker for detecting autonomic and inflammatory changes in COVID-19 and post–COVID-19 condition, with studies linking reduced HRV indexes with infection dynamics and persistent symptoms [37,39,41,42,49,53]. The integration of wearable devices with Internet of Things advancements has enabled noninvasive HRV data collection, expanding opportunities for remote health monitoring [54]. However, previous research has faced several challenges, including small sample sizes, demographic homogeneity, variations in HRV measurement techniques, and the lack of standardized data collection protocols. Moreover, many studies have relied on traditional statistical analyses rather than predictive modeling, limiting their ability to capture complex HRV patterns associated with COVID-19 and post–COVID-19 condition. To address these gaps, further research is needed to consolidate findings and refine the understanding of HRV alterations in COVID-19 and post–COVID-19 condition. The review presented in these sections examines key studies on these relationships, highlighting trends, limitations, and open questions in the field.

#### HRV, COVID-19, and Wearable Devices

Recent studies have explored the use of wearable devices in analyzing HRV in relation to SARS-CoV-2 infection. One case report described reduced HRV in a male patient aged 52 years confirmed through RT-PCR, suggesting autonomic imbalance, although preexisting obesity and hypertension may have influenced the result [55]. Another study found that significant HRV reductions preceded increases in CRP in hospitalized patients, pointing to HRV as a possible early indicator of inflammatory exacerbation. However, the study lacked a control group and had a high prevalence of comorbidities, including cardiovascular disease in 71% of participants [32].

Machine learning models have been used to analyze HRV data for COVID-19 detection. One study used self-reported data from 186 participants and achieved 83% accuracy in detecting infection 2 days before symptom onset [56]. Nonetheless, the lack of confirmed diagnoses and wide variability in BMI, age, sex, climate exposure, and device types (Fitbit, Garmin, and Apple Watch) undermines its generalizability.

A logistic regression model compared physiological data from 14 individuals with COVID-19 and 14 healthy controls before and after physical activity, revealing reduced HRV in the infected group after activity. However, the study’s generalizability is limited by significant demographic differences between groups, particularly differences in age and BMI [57].

Changes in circadian HRV amplitude were detected via Apple Watch up to 7 days before diagnosis in health care workers who tested positive for COVID-19 regardless of symptoms, suggesting predictive potential [43]. However, this study’s statistical approach (mixed-effects cosinor models) may have missed subtle nonlinear patterns.

Continuous monitoring of 2 pregnant women during SARS-CoV-2 infection confirmed through RT-PCR revealed a reduction in HRV indexes over 3 to 6 days at the peak of physiological alterations followed by a return to baseline levels after recovery. While this study highlights the potential of wearable devices for tracking physiological changes during pregnancy, its findings are limited by the small sample size [58]. Another study using a recurrent neural network–long short-term memory model (RNN-LSTM) with Ava bracelet data identified infection 2 days before symptom onset in 68% of cases, but it excluded asymptomatic individuals and focused on participants aged <51 years [44].

A study on HRV and COVID-19 achieved an area under the curve (AUC) of 0.77 for predicting infection using a convolutional neural network (CNN) trained on *Z* score–transformed data (respiratory rate, heart rate, root mean square of successive differences [RMSSD], and entropy) over 5 days. The CNN–based model lacked the ability to differentiate COVID-19 from other respiratory infections such as influenza, potentially reducing its specificity [21]. Another study identified differences in HRV variation at symptom onset between male and female individuals using statistical methods [59]. While both of these studies leveraged wearable devices and large datasets, they faced limitations due to unverified self-reported infection status.

A study classified COVID-19 presence and severity with 94% accuracy using random forest and support vector machine (SVM) models trained on HRV parameters from photoplethysmographic signals comparing 50 healthy participants to 93 older patients with mild to moderate disease [37]. It distinguished healthy individuals from mild cases (94%) and mild from moderate cases (89%). While demonstrating strong performance using wearable-derived features, its generalizability is limited by a single-center cohort, demographic imbalances in age and sex, and unreported BMI.

#### HRV and Post–COVID-19 Condition

Some studies suggest that post–COVID-19 condition may be associated with dysautonomia, reflected in changes in HRV. Prolonged symptoms have been proposed to result from this dysautonomia, as highlighted in the study by Dani et al [60]. Patients with post–COVID-19 condition and fatigue exhibit HRV dysregulation, as shown in the study by Barizien et al [53], suggesting HRV as a potential neuroimmune link to this condition. Significant reductions in HRV indexes, including low-frequency (LF) and high-frequency (HF) components, were reported in the studies by Aranyó et al [47] and Marques et al [49] among patients with post–COVID-19 condition compared to recovered individuals and healthy controls, highlighting global HRV impairment. Reduction in the SD of the normal-to-normal intervals (SDNN), RMSSD, and HF components was observed in 30 hospitalized patients with post–COVID-19 condition compared to 20 controls using 24-hour electrocardiography (ECG) [46].

Reductions in RMSSD were also observed in patients after COVID-19, with partial recovery over 6 months, particularly among those hospitalized in the intensive care unit [48]. Additional studies [50,52] have confirmed reductions in HRV indexes, including SDNN and RMSSD, in patients with post–COVID-19 condition compared to healthy controls. These studies also reported shifts in frequency-domain metrics such as increased LF and decreased HF components, reflecting potential autonomic imbalances.

Reductions in HRV across multiple metrics, including SDNN and LF components, have also been observed in patients after COVID-19, as reported in the studies by Mooren et al [51] and Liu et al [61]. Reduction in SDNN and SDNN index with an increased LF-to-HF ratio was observed in 87 patients following acute COVID-19 using 24-hour Holter ECG before and within 3 months after RT-PCR confirmation [62]. A case-control study [63] assessed resting HRV and HRV during deep breathing maneuvers (maneuver of accentuation of respiratory sinus arrhythmia [M-RSA]) in 21 patients with post–COVID-19 condition and 20 controls. At rest, patients with post–COVID-19 condition exhibited significant reductions in several HRV indexes alongside an elevated mean heart rate, suggesting autonomic dysfunction.

A systematic review [64] analyzed 17 observational studies (N=3628) investigating the impact of SARS-CoV-2 infection on HRV compared to healthy controls. RMSSD emerged as the most frequently evaluated parameter; reductions in RMSSD and HF components were associated with infection or linked to COVID-19 severity, poor prognosis, and mortality in patients who were critically ill. Collectively, these studies highlight HRV’s growing role in advancing our understanding of its alterations in post–COVID-19 condition and suggest its potential to differentiate patients after COVID-19 from healthy controls. Despite methodological variability, they consistently establish HRV as a valuable marker of physiological changes.

#### Research Gaps and Study Contribution

Although significant progress has been made in using HRV data to understand the physiological impacts of COVID-19 and post–COVID-19 condition, several key gaps remain unaddressed. Many retrospective studies rely on precollected datasets with self-reported SARS-CoV-2 infections, limiting their accuracy and applicability. Furthermore, most studies use traditional statistical methods, which, while useful, are limited in their ability to uncover complex HRV patterns that may distinguish individuals who are infected, patients after COVID-19, and healthy controls.

A fundamental gap in the current literature is the lack of well-defined HRV patterns across these 3 groups. While previous research has identified reductions in certain HRV indexes, there is no consensus on which specific metrics reliably indicate SARS-CoV-2 infection or long-term autonomic dysfunction. Variability in data collection methods, ranging from consumer-grade wearables to clinical ECG monitors, further introduces inconsistencies that complicate comparative analyses. To address these gaps, this study proposes a machine learning–driven approach to identify distinct HRV patterns among 61 participants comprising individuals with active COVID-19 and post–COVID-19 condition and healthy individuals using wearable-derived clinical data and publicly available sources, incorporating previous infection history when available to enhance predictive accuracy. These established HRV patterns not only enable the classification of patient conditions in existing datasets but also serve as the foundation for a pioneering near–real-time health monitoring system validated on 4 participants. This approach enhances predictive accuracy and supports early detection and continuous management of COVID-19 and its long-term effects, as detailed in the following section.

### Objectives

This study aimed to identify distinct HRV patterns capable of differentiating among individuals with active COVID-19 and post–COVID-19 condition and healthy controls using data obtained from wearable devices. By applying supervised machine learning to HRV indexes, we sought to classify physiological states based on autonomic and inflammatory signatures. A secondary objective was to evaluate the technical feasibility of implementing a near–real-time health monitoring system that integrates HRV signal acquisition, preprocessing, and classification into an automated workflow for continuous, noninvasive assessment of health status.

## Methods

### Overview

This section outlines the data sources, preprocessing methods, HRV indexes, and machine learning techniques used to delineate distinctive HRV patterns in healthy individuals, patients with COVID-19, and those with post–COVID-19 condition. By integrating clinical and public datasets, we sought to establish physiological signatures of SARS-CoV-2 infection and its persistent sequelae applicable to both existing records and prospective data collection. We further demonstrate a custom-built system for near–real-time HRV monitoring, highlighting its potential as an innovative application of these findings.

### Study Dataset

This study analyzed HRV data from 3 distinct groups: healthy individuals (20/61, 33%), patients with active COVID-19 (21/61, 34%), and those with post–COVID-19 condition (20/61, 33%). In total, 2 datasets were integrated: a clinical dataset from Professora Lydia Storópoli Hospital, São Paulo, Brazil, and a public dataset from the Fit-COVID Study [65], a cross-sectional observational investigation. The demographic and clinical profiles of these groups are summarized in Table 1, with post–COVID-19 condition symptoms reported by the participants and comorbidities detailed in Table 2. The Fit-COVID dataset provided HRV data from 20 healthy individuals who tested negative for COVID-19 via immunoglobulin M or immunoglobulin G antibody tests, as well as 20 individuals with post–COVID-19 condition who had a confirmed polymerase chain reaction positive COVID-19 diagnosis, exhibited mild to moderate symptoms, and were assessed 15 to 180 days after infection. All participants were aged 20 to 40 years [65].

**Table 1:** Demographic and clinical characteristics of the individuals from the datasets used.

Feature			COVID-19 (n=21)		Healthy (n=20)		Post–COVID-19 condition (n=20)
**Sex, n (%)**							
	Female	10 (48)		10 (50)		9 (45)	
	Male	11 (52)		10 (50)		11 (55)	
Age (y), mean (SD)			49 (18)		26 (5)		29 (6.3)
Weight (kg), mean (SD)			84 (16)		74 (16)		76 (14)
Height (cm), mean (SD)			166 (8)		173 (9)		170 (9)
BMI (kg/m^2^), mean (SD)			30 (5)		24.8 (4.7)		26.7 (5)
SBP^a^ (mm Hg), mean (SD)			124 (21)		120 (5.6)		127 (21)
DBP^b^ (mm Hg), mean (SD)			80 (15)		80 (2)		80 (4)
Symptom onset (d), mean (SD)			6 (2)		—^c^		—

^a^SBP: systolic blood pressure.

^b^DBP: diastolic blood pressure.

^c^Not applicable.

**Table 2:** Symptom prevalence in participants with post–COVID-19 condition (N=20).

Symptom	Participants, n (%)
Respiratory problems	15 (75)
Body aches	14 (70)
Headache	14 (70)
Anosmia (loss of smell)	10 (50)
Ageusia (loss of taste)	9 (45)
Fever	6 (30)
Diarrhea	5 (25)

Exclusion criteria included chronic diseases, smoking, recent vaccination, or medications affecting autonomic function. A general anamnesis, BMI, and physical activity levels were evaluated. All HRV recordings in the Fit-COVID Study were conducted in the morning to minimize the effects of circadian variation. Participants were instructed to abstain from physical activity, caffeine, and alcohol for 24 hours before data collection to reduce external influences on autonomic function. Measurements were taken in a quiet, controlled environment, with participants seated and breathing spontaneously. After a 20-minute rest period, RR intervals were recorded for 25 minutes using a Polar RS800CX device with a 1-kHz sampling rate. HRV indexes were subsequently extracted using the Kubios HRV software (Kubios Oy). The clinical dataset comprised 45 hospitalized patients with active COVID-19 (RT-PCR confirmed, moderate to severe symptoms, and onset of 1-10 days) collected in October 2022. Common comorbidities among these patients included hypertension (21/45, 47%), diabetes (12/45, 27%), obesity (19/45, 42%), chronic obstructive pulmonary disease (5/45, 11%), and smoking (1/45, 2%), with 66% (30/45) vaccinated (predominantly with CoronaVac; 22/45, 49% first dose and 16/45, 36% second dose). During their hospital stay, treatments frequently included antibiotics (37/45, 83%), corticosteroids (42/45, 94%), anticoagulants (40/45, 88%), and antihypertensives (41/45, 90%). To ensure consistency and reduce measurement bias, all HRV recordings for the COVID-19 group were performed under standardized conditions during hospitalization. Each patient remained in a quiet, temperature-controlled room and was instructed to rest in the supine position for 20 minutes before data collection. HRV data were then recorded for 15 minutes using a Polar V800 device at a sampling rate of 1 kHz.

Patients were asked to remain still and silent throughout the session to minimize artifacts. Data were collected during daytime hours, typically in the morning, to reduce the influence of circadian variations on autonomic function. This protocol followed the established recommendations for short-term HRV measurement [66]. Single HRV recording was performed per participant under these controlled conditions as repeat measurements were not feasible in the clinical setting. Even under these controlled conditions, the collected RR interval signals exhibited discrepancies, potentially caused by voluntary or involuntary movements, acquisition failures, or conditions such as excessive sweating.

To ensure data quality, preprocessing steps were applied using the method by Tukey [67] for outlier detection and filtering, with thresholds determined by the IQR, defined as the difference between the third quartile and the first quartile. The following thresholds were used for identifying outliers: first quartile − 2.0 × IQR for the lower limit and third quartile + 2.0 × IQR for the upper limit.

The sensitivity factor *K*=2.0 was chosen to enhance precision in detecting artifacts and outliers. Despite these efforts, 53% (24/45) of the participants still exhibited significant artifacts or unstable signals and were excluded from the analysis. In this hospitalized population, repeating the HRV measurement was not always feasible due to logistical and clinical constraints. The remaining signals were reassessed using the Kubios software to confirm stability, resulting in 47% (21/45) of the participants providing at least 10 minutes of stable signals, enabling the extraction of HRV indexes.

Unlike several previous studies that relied on physiological data collected under minimally controlled conditions, this study used data collected in accordance with established recommendations for short-term HRV analysis [66], applying standardized protocols across 2 distinct datasets—hospitalized patients in the clinical cohort and outpatient volunteers in the observational one. Both adhered to core methodological practices, including standardized posture, prerecording rest, spontaneous breathing, daytime data acquisition, and quiet environments. This uniformity in data collection helped minimize procedural bias and enhanced the reliability and comparability of the extracted HRV indexes across groups.

The data in Tables 1 and 2, together with the comorbidity information of individuals who were infected described throughout this section, characterize the study cohorts, with the selection of and rationale for the HRV indexes used to identify physiological markers of SARS-CoV-2 infection detailed in the following section.

### Variables Selected

To identify physiological markers of SARS-CoV-2 infection and its sequelae, we selected HRV indexes from the Fit-COVID and clinical datasets guided by their physiological relevance and established associations with COVID-19 pathology.

We focused on linear methods using time-domain indexes, specifically SDNN and RMSSD as they measure overall HRV and short-term parasympathetic activity, respectively, with SDNN reflecting global variability that decreases with age [66,68]. RMSSD is a reliable index of HRV modulation by vagal activity, as demonstrated in previous studies [66,69,70]. In the frequency domain, we used relative power in LF and HF bands, representing sympathovagal balance, which is less influenced by baseline heart rate and aging compared to absolute power, increasing comparability and generalizability across populations.

Previous studies involving COVID-19 and post–COVID-19 condition have explored absolute power variations in LF and HF measured in milliseconds squared, identifying reductions in these indexes. However, this approach may introduce bias as absolute power is influenced by the individual’s basal heart rate [66] and significantly decreases with aging [71].

Frequency-domain analyses based on absolute power exhibit higher sensitivity than those using relative power [72,73], but relative power mitigates these biases, enhancing cross-study comparability and generalizability, particularly for short-term recordings in populations with COVID-19. Nonlinear methods such as Poincaré plot SD perpendicular to the line of identity 'SD1 and SD2 along the line of identity were excluded due to the lack of established associations with COVID-19 in the literature.

The selection was guided by scientific evidence linking SDNN to overall HRV, RMSSD to parasympathetic modulation, and LF and HF relative power to autonomic dysregulation, all of which are implicated in the inflammatory and cardiovascular responses of SARS-CoV-2 infection and post–COVID-19 condition. We selected LF and HF relative power over absolute power or normalized units to mitigate biases associated with basal heart rate, aging, and interindividual variability, as well as to reduce susceptibility to artifacts in short-term HRV assessments. This choice addresses a gap in the literature, where studies on COVID-19 and post–COVID-19 condition predominantly report absolute or normalized power but rarely relative power despite its advantages for detecting autonomic dysregulation in inflammatory conditions and other ANS anomalies.

The use of LF and HF relative power enhances comparability across diverse populations—including healthy controls, patients with COVID-19, and survivors of post–COVID-19 condition—by being independent of basal heart rate, thereby improving generalizability [66,72-75]. In addition, LF and HF relative power facilitates interpretation and comparison between studies and individuals.

The selected variables (SDNN, RMSSD, and LF and HF relative power) were organized into a CSV file for input into machine learning tools, as detailed in the next section. Statistical analyses, including the Kolmogorov-Smirnov test to assess normality, were conducted. For nonnormally distributed data, the nonparametric Kruskal-Wallis and Mann-Whitney *U* tests (*P*<.05) were applied to validate the distribution of these indexes, with results visualized via box plots to assess variability and outliers. These analyses, presented in the Results section, support the validity of our variable selection for distinguishing physiological states across these groups.

To ensure that the observed differences between the groups were not confounded by demographic factors such as age and BMI, we applied statistical analysis before implementing machine learning algorithms.

### Statistical Analysis

Analysis of covariance (ANCOVA) was used to control for potentially confounding effects related to the higher age and BMI observed in the infected group compared to the other groups, as well as their influence on HRV variables. ANCOVA is a statistical technique that combines ANOVA and linear regression to adjust for the effects of continuous covariates while examining the influence of the categorical independent variable on the dependent variable [76].

In this study, the COVID-19 group showed significant differences in age and BMI compared to the other groups. Studies indicate that advanced age is associated with a reduction in HRV both in short-term monitoring and in 24-hour recordings [68,77]. In particular, age negatively influences HRV after high-intensity exercise, especially in older individuals [78]. Furthermore, the relationship between age and HRV has also been observed in patients with chronic headache [79].

Similarly, BMI is often associated with reduced HRV, especially in individuals with obesity or metabolic syndrome. However, studies indicate that BMI alone may not be a robust predictor of HRV when compared to more specific measures of adiposity. One study [80] found that, while BMI alone did not show a significant correlation with HRV indexes, central adiposity, measured through waist circumference, was more strongly correlated with reduced HRV. Another study demonstrated that central adiposity has a more direct impact on HRV than overall BMI [81].

The ANCOVA model included HRV indexes (SDNN, RMSSD, and LF and HF relative power) as dependent variables, the infection group as a categorical factor, and age and BMI as continuous covariates. The assumption of homogeneity of slopes was assessed to ensure that the relationship between the covariates (age and BMI) and the dependent variable did not vary between groups.

Before the main modeling, we conducted several tests to verify the assumptions of the ANCOVA. The Pearson correlation was used to assess the relationship between age and HRV, as well as between BMI and HRV. An interaction test (group × covariate) was conducted to confirm the homogeneity of slopes, whereas the Shapiro-Wilk test ensured the normality of residuals. To verify the homogeneity of variances between groups, the Levene test was applied. This methodological approach allowed us to assess both the direct impact of COVID-19 on HRV and the potential interaction between the covariates (age and BMI) and the analyzed groups.

### Algorithms and Training

To optimize the classification algorithm for the dataset, the machine learning and deep learning application in MATLAB (version R2023b, Student Edition; The MathWorks, Inc) was used. This platform tested multiple classifiers—decision trees, SVMs, k-nearest neighbor (k-NN), artificial neural networks, and ensemble methods—with automated hyperparameter tuning and cross-validation. The HRV indexes SDNN, RMSSD, and LF and HF relative power, sourced from clinical and public datasets and extracted using the Kubios software, were used to evaluate algorithm performance.

In total, 2 training rounds were conducted: the first used only the 4 HRV indexes, and the second included an additional contextual predictor, *covidrecent*. This temporal variable was coded as 1 only for participants in the post–COVID-19 condition group and 0 for all others (those with acute infection and healthy) based on patient-reported SARS-CoV-2 infection within the previous 3 months. This criterion mimics real-world triage scenarios where recent infection history is typically available during intake interviews and may aid diagnostic refinement. It was not derived from the output label but rather represents a realistic feature used to enhance model performance in triage scenarios.

The full set of input values used in this phase, including HRV indexes and the *covidrecent* variable, is available in Multimedia Appendix 1. A 5-fold stratified cross-validation approach ensured robust validation across the 61-participant dataset (n=21, 34% infected; n=20, 33% healthy; and n=20, 33% with post–COVID-19 condition).

The classification accuracy served as the primary evaluation metric during this phase; however, precision, recall, and *F*_1_-score were also considered to gain a comprehensive understanding of algorithm performance. Hyperparameter tuning was performed automatically using MATLAB’s built-in optimization functions to tailor the algorithms to the datasets’ characteristics and enhance predictive performance. Specific hyperparameters adjusted for each model family are detailed in Multimedia Appendix 1, along with accuracy values before and after optimization. The results were ranked based on classification accuracy obtained during validation tests, providing an initial indication of the most suitable models for further refinement.

Following the identification of the most promising models in MATLAB, these algorithms were reimplemented in Python (Python Software Foundation) using the scikit-learn library. During the transition to Python, validation tests were conducted to ensure consistency between the models’ performance in MATLAB and Python. This transition enabled greater flexibility for additional experimentation, facilitated integration with other commonly used machine learning frameworks, and supported the development of a near–real-time prediction solution.

In MATLAB, 5-fold stratified cross-validation was initially applied. For the Python implementation, a 15-fold k-fold stratified strategy was adopted to enhance the robustness of performance estimates. While increasing the number of folds reduces the training set per iteration, it provides more stable aggregated metrics. A fixed random seed (random state=42) was used to ensure full reproducibility across all model training and validation steps.

### Near–Real-Time Data Acquisition and Analysis

To assess the feasibility of near–real-time physiological monitoring based on HRV, we developed a custom-designed fingertip pulse oximeter capable of collecting photoplethysmography (PPG) signals and transmitting them to a server using the MQTT protocol. The raw PPG data were stored in a MySQL database and processed using a Python-based pipeline to extract HRV indexes, SDNN, RMSSD, and LF and HF relative power in both the time and frequency domains.

HRV computation was conducted using the HeartPy library [82], which applies preprocessing routines, including outlier rejection, peak detection, and filtering. Before HRV extraction, PPG signals were normalized using minimum to maximum scaling and processed using a second-order Butterworth band-pass filter (0.5-3.5 Hz) to minimize baseline wander and HF noise. A minimum recording duration of 2 minutes was adopted for each session in accordance with established recommendations for LF HRV assessment.

The system was designed to automatically transmit HRV features at the end of each recording cycle, enabling continuous physiological monitoring without user intervention. A web-based interface displays the classified physiological state—COVID-19, post–COVID-19 condition, or healthy—based on previously trained machine learning models.

**Figure 1:**
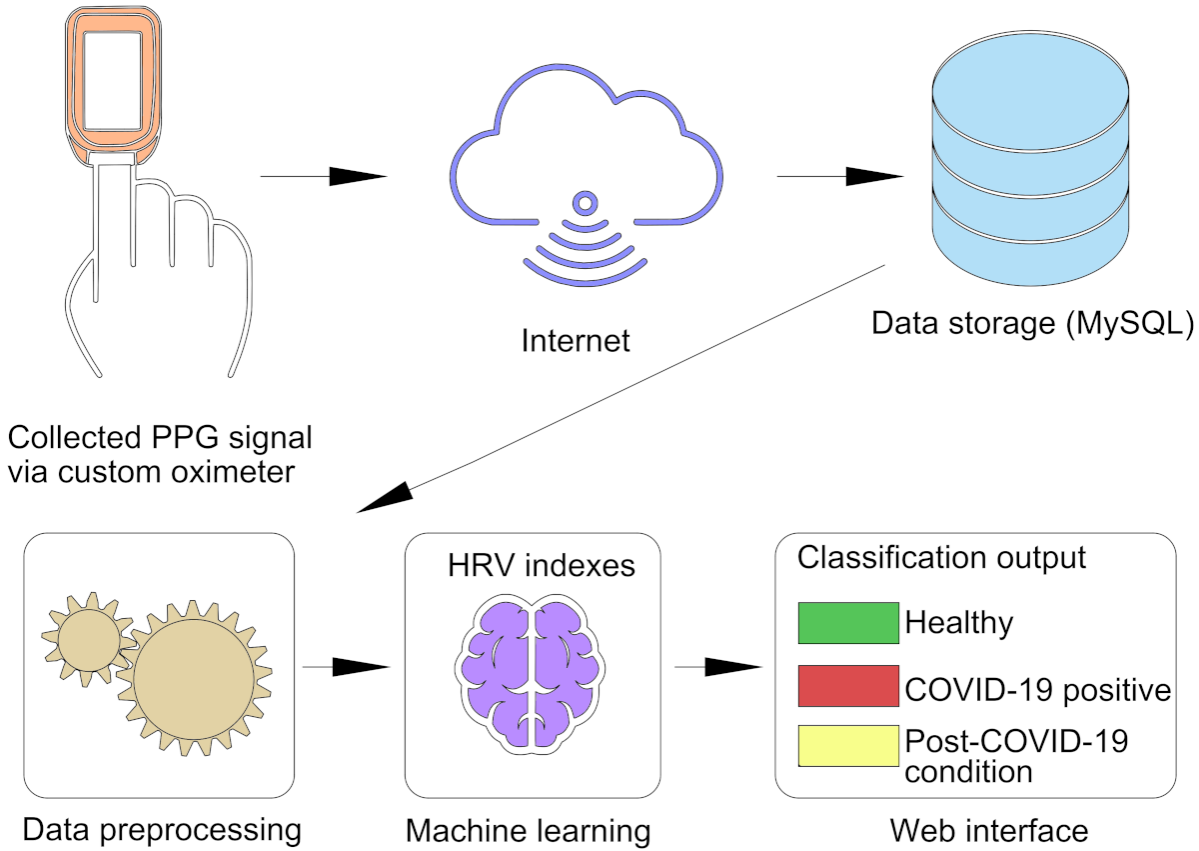
Near–real-time health monitoring system workflow. HRV: heart rate variability; PPG: photoplethysmography.

A feasibility demonstration was conducted with 4 independent participants: 2 (50%) healthy individuals and 2 (50%) individuals with RT-PCR–confirmed active COVID-19. Each participant underwent a 2-minute recording session, after which the system completed signal preprocessing, HRV computation, and classification in approximately 1 second. Figure 1 illustrates the overall workflow of data acquisition, processing, and classification.

This application demonstrates that a fully functional, autonomous system can perform near–real-time HRV-based classification of physiological states. While the system itself is operational, the robustness and generalizability of the physiological signatures used for classification would benefit from validation in larger and more diverse populations. The implications and limitations of these findings are further discussed in the Limitations section.

Additional technical details regarding the hardware architecture, signal processing, and transmission protocols are available in Multimedia Appendix 2.

### Ethical Considerations

This study used data from 3 ethically approved sources, all in accordance with Brazilian Resolution 466/12 of the National Health Council and the Declaration of Helsinki.

The first dataset was obtained from a previously approved clinical study involving patients with COVID-19 conducted under protocol 46699521.5.0000.5511 and approved by the research ethics committee of Nove de Julho University.

The second dataset was sourced from the publicly available Fit-COVID Study, a cross-sectional observational investigation conducted under independent ethics approval. This dataset includes anonymized HRV recordings from healthy individuals and patients with post–COVID-19 condition, and its use in this study complied with ethical reuse and secondary data use guidelines.

The third dataset was collected by the authors under protocol 68909623.0.0000.5511, also approved by the research ethics committee of Nove de Julho University. It involved near–real-time HRV index acquisition across different clinical stages.

All participants were aged >18 years and provided written informed consent.

All data were anonymized before analysis, and HRV recordings were acquired following standardized conditions for short-term HRV analysis. This study complied with all applicable regulations concerning human participant research and data protection.

## Results

The HRV indexes SDNN, RMSSD, and LF and HF relative power were extracted from the RR intervals of 21 participants actively infected with SARS-CoV-2, a total of 20 healthy participants, and 20 participants with post–COVID-19 condition, totaling 61 individuals. These data, which constitute the core dataset for identifying HRV patterns associated with each health status, were compiled for algorithm training and are summarized in Table 3.

**Figure 2:**
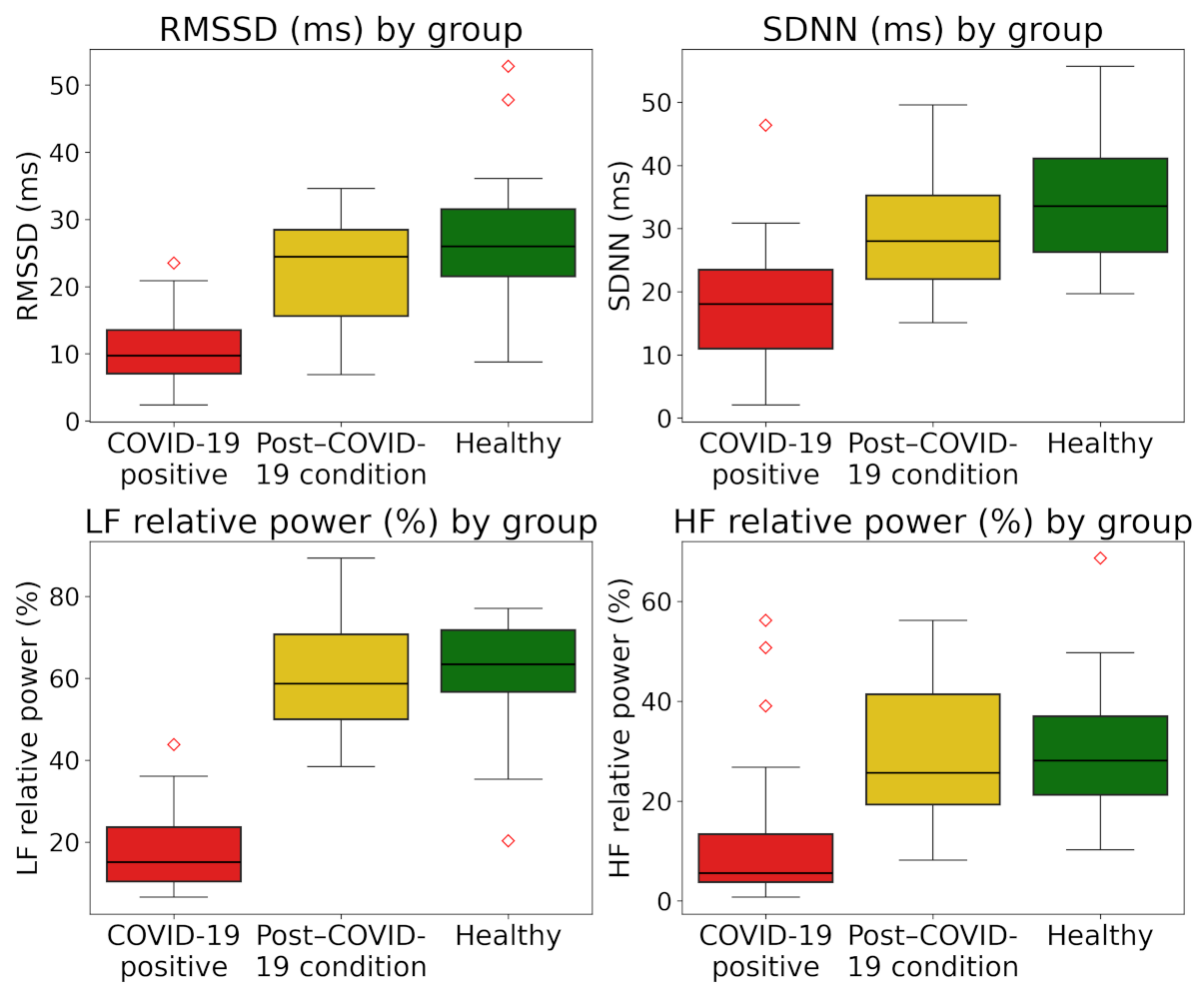
Box plot of heart rate variability indexes across participant groups. HF: high frequency; LF: low frequency; RMSSD: root mean square of successive differences; SDNN: SD of the normal-to-normal intervals.

The HRV indexes presented in Table 3 reveal distinct patterns among the 3 groups: healthy participants, those with post–COVID-19 condition syndrome, and those actively infected with SARS-CoV-2. These data were further visualized using box plots to examine distribution and variability of HRV indexes, as shown in Figure 2.

Figure 2 indicates that, for the group with COVID-19, all HRV indexes (SDNN, RMSSD, and LF and HF relative power) were consistently lower than those of the healthy and post–COVID-19 condition groups. In contrast, the differences between the healthy and post–COVID-19 condition groups were subtler, particularly in the frequency-domain indexes (LF and HF relative power), where overlap in distributions was observed.

To statistically evaluate these differences, the Kruskal-Wallis test was applied to HRV indexes. After the Kolmogorov-Smirnov test confirmed the nonnormality of the data, pairwise comparisons using the Mann-Whitney *U* test with a significance level of *P*<.05 were conducted. The results of these analyses are presented in Tables 4 and 5.

**Table 3:** Indexes for participants actively infected with SARS-CoV-2, participants with post–COVID-19 condition, and healthy controls.

Groups and participants		RMSSD^a^	SDNN^b^	LF^c^ relative power	HF^d^ relative power
**COVID-19 group**					
	1	9.7	16.1	23.6	6.8
	2	9.7	23.5	19.1	3.2
	3	14.0	21.1	23.9	39.0
	4	10.7	15.5	43.8	8.9
	5	13.2	18.3	16.7	26.8
	6	16.4	24.0	19.3	4.6
	7	3.9	9.3	13.6	3.8
	8	14.1	25.8	11.6	5.5
	9	10.8	19.9	11.6	5.6
	10	13.6	18.1	30.9	24.1
	11	20.9	26.2	6.6	50.7
	12	3.1	10.9	15.1	1.6
	13	8.3	11.0	24.0	13.3
	14	23.5	18.2	16.3	56.2
	15	5.5	9.7	10.4	7.6
	16	9.0	46.3	8.6	0.8
	17	2.4	2.1	7.8	13.2
	18	10.5	13.9	7.5	4.4
	19	4.5	10.5	11.4	1.1
	20	7.0	30.9	7.3	2.3
	21	7.8	15.9	36.1	5.2
**Post–COVID-19 condition group**					
	1	10.7	18.4	89.3	8.3
	2	24.7	32.4	76.7	22.0
	3	17.8	27.9	71.9	21.2
	4	34.1	43.1	55.4	37.5
	5	21.2	28.1	38.4	56.2
	6	16.2	22.8	61.9	31.0
	7	6.9	15.6	59.0	9.6
	8	13.8	19.6	80.1	18.5
	9	17.9	26.9	49.1	29.3
	10	7.2	15.1	85.2	8.2
	11	9.7	16.7	66.7	16.6
	12	34.6	30.9	49.5	48.3
	13	30.6	49.6	70.3	21.0
	14	27.6	33.8	50.1	45.9
	15	31.0	38.3	58.4	38.6
	16	28.6	37.0	66.4	19.6
	17	26.7	27.8	41.7	19.9
	18	28.4	35.0	51.0	43.2
	19	24.2	36.0	52.3	40.7
	20	27.3	27.6	44.7	49.1
**Healthy controls**					
	1	25.6	33.4	73.0	21.8
	2	21.2	30.2	71.4	26.2
	3	25.5	33.4	75.7	19.3
	4	31.2	22.1	49.4	37.6
	5	52.8	55.7	54.3	37.0
	6	36.1	44.1	60.7	37.0
	7	24.8	33.7	77.0	17.5
	8	26.4	36.6	57.4	35.0
	9	34.8	37.4	20.3	68.6
	10	8.8	19.7	62.6	14.3
	11	20.8	26.1	76.5	21.7
	12	24.0	26.3	60.8	37.8
	13	21.6	39.4	68.2	10.2
	14	28.4	41.3	64.2	23.9
	15	20.7	24.6	67.8	26.0
	16	17.6	22.4	76.6	19.9
	17	30.0	45.5	50.7	35.9
	18	47.8	53.4	68.7	30.0
	19	32.6	41.0	59.2	35.1
	20	29.4	26.9	35.4	49.7

^a^RMSSD: root mean square of successive differences.

^b^SDNN: SD of the normal-to-normal intervals.

^c^LF: low frequency.

^d^HF: high frequency.

**Table 4:** Heart rate variability indexes for different groups.

Index	Infected, mean (SD)	Post–COVID-19 condition, mean (SD)	Healthy, mean (SD)
SDNN^a^	18.42 (9.38)	29.13 (9.37)	34.66 (10.2)
RMSSD^b^	10.39 (5.51)	21.96 (8.88)	28 (9.91)
LF^c^ relative power	17.38 (10.04)	60.89 (14.54)	61.5 (14,49)
HF^d^ relative power	13.54 (16.41)	29.23 (14.78)	30.21 (13.37)

^a^SDNN: SD of the normal-to-normal intervals.

^b^RMSSD: root mean square of successive differences.

^c^LF: low frequency.

^d^HF: high frequency.

**Table 5:** Kruskal-Wallis and Mann-Whitney statistical tests.

HRV^a^ index and group comparison		*U*^b^ statistic	Mann-Whitney test *P* value	Effect size (*r*)^c^	Kruskal-Wallis *H*		Kruskal-Wallis test *P* value	
**SDNN^d^**						22.245		<.001
	Healthy vs COVID-19	377	<.001	0.680				
	Healthy vs post–COVID-19 condition	254	.14	0.233				
	COVID-19 vs post–COVID-19 condition	78	.001	0.538				
**RMSSD^e^**						27.815		<.001
	Healthy vs COVID-19	399	<.001	0.770				
	Healthy vs post–COVID-19 condition	257	.13	0.244				
	COVID-19 vs post–COVID-19 condition	64	<.001	0.595				
**LF^f^** **relative power**						38.847		<.001
	Healthy vs COVID-19	412	<.001	0.823				
	Healthy vs post–COVID-19 condition	222	.56	0.094				
	COVID-19 vs post–COVID-19 condition	2	<.001	0.847				
**HF^g^ relative power**						16.079		<.001
	Healthy vs COVID-19	344	.001	0.546				
	Healthy vs post–COVID-19 condition	206	.88	0.026				
	COVID-19 vs post–COVID-19 condition	80	.001	0.530				

^a^HRV: heart rate variability.

^b^Mann-Whitney *U* statistic.

^c^Effect size based on *Z*/√N.

^d^SDNN: SD of the normal-to-normal intervals.

^e^RMSSD: root mean square of successive differences.

^f^LF: low frequency.

^g^HF: high frequency.

Table 4 provides the descriptive statistics for each HRV index across groups. To assess the significance and magnitude of these differences, Table 5 reports the results of nonparametric statistical tests and corresponding effect sizes.

The 95% CIs for the effect sizes were estimated using the Fisher *Z* transformation and are reported in Multimedia Appendix 1.

The Kruskal-Wallis test revealed significant differences among the 3 groups for all HRV indexes (*P*<.001). Mann-Whitney *U* tests confirmed that these differences were statistically significant only between the infected group and the other 2 groups, with large effect sizes (*r*≥0.5). Comparisons between the healthy and post–COVID-19 condition groups did not reach significance (*P*>.12), with small effect sizes (*r*<0.3), indicating that overt group-level differences were not detected in isolated HRV features. The effect size *r* (calculated as *Z*/√N) provides a standardized estimate of the magnitude of group differences, offering additional interpretability beyond *P* values.

These findings underscore the limitations of traditional statistical methods in capturing nuanced autonomic changes, particularly between individuals with post–COVID-19 condition and healthy individuals, thus motivating the application of more sophisticated analytical approaches.

Our machine learning approach distinguished participants with post–COVID-19 condition from healthy participants, indicating the presence of subtle, multidimensional patterns in short-term HRV data that are not readily detected by conventional statistical tests. This highlights the importance of integrating physiological insights with advanced computational methods to identify latent autonomic dysregulation in post–COVID-19 conditions.

To address potential confounding variables such as age and BMI, which may influence HRV independently of infection status, we applied propensity score matching (PSM) to create balanced subgroups before further statistical analysis. PSM was implemented using logistic regression to estimate propensity scores based on age and BMI followed by nearest neighbor matching using a caliper of 0.3. The resulting subset comprised 15 participants (n=5, 33% per group) with comparable age and BMI: healthy group (mean age 25.33, SD 4.96 years; mean BMI 23.01, SD 4.52 kg/m^2^), post–COVID-19 condition group (mean age 26.22, SD 3.87 years; mean BMI 22.76, SD 2.16 kg/m^2^), and infected group (mean age 29.60, SD 7.40 years; mean BMI 28.51, SD 5.10 kg/m^2^). Compared to the original cohort, this subset exhibited lower BMI values, likely due to the initial imbalance in BMI distribution favoring higher values in the infected group.

To assess group differences in HRV indexes (SDNN, RMSSD, and LF and HF relative power) while accounting for residual effects of age and BMI, we then conducted an ANCOVA incorporating infection group as a categorical factor and age and BMI as continuous covariates. The assumptions of homogeneity of variances (Levene test) and normality of residuals (Shapiro-Wilk test) were verified and satisfied. The results of this model are summarized in Table 6, which presents the adjusted comparisons of HRV indexes across the matched groups after controlling for age and BMI. Table 7 shows the verification of the assumptions of homogeneity of variances (Levene test) and normality of residuals (Shapiro-Wilk test).

**Table 6:** Analysis of covariance results for heart rate variability (HRV) indexes after controlling for age and BMI.

HRV index and post hoc comparison			Group effect *P* value		Group effect, *F* test (*df*)		Post hoc *P* value		Age effect *P* value		BMI effect *P* value
**SDNN^a^**					2.4691 (2, 10)		.13		.45		.94
	Infected vs healthy	.04									
	Infected vs post–COVID-19 condition	.77									
	Healthy vs post–COVID-19 condition	.01									
**RMSSD^b^**					4.9757 (2, 10)		.03		.52		.40
	Infected vs healthy	.02									
	Infected vs post–COVID-19 condition	.44									
	Healthy vs post–COVID-19 condition	.01									
**LF^c^ relative power**					12.5032 (2, 10)		.002		.21		.90
	Infected vs healthy	.003									
	Infected vs post–COVID-19 condition	.20									
	Healthy vs post–COVID-19 condition	.003									
**HF^d^ relative power**					0.8544 (2, 10)		.45		.64		.40
	Infected vs healthy	.25									
	Infected vs post–COVID-19 condition	.31									
	Healthy vs post–COVID-19 condition	.07									

^a^SDNN: SD of the normal-to-normal intervals.

^b^RMSSD: root mean square of successive differences.

^c^LF: low frequency.

^d^HF: high frequency.

**Table 7:** Assumption verification—normality and homogeneity of variances.

HRV^a^ index	Normality (Shapiro-Wilk test), *P* value	Homogeneity (Levene test), *P* value
SDNN^b^	.50	.79
RMSSD^c^	.48	.70
LF^d^ relative power	.99	.95
HF^e^ relative power	.34	.91

^a^HRV: heart rate variability.

^b^SDNN: SD of the normal-to-normal intervals.

^c^RMSSD: root mean square of successive differences.

^d^LF: low frequency.

^e^HF: high frequency.

Table 6 presents the ANCOVA results for HRV indexes after adjusting for age and BMI with post hoc comparisons. *P* values for SDNN and HF relative power should be interpreted cautiously due to nonsignificant group effects (*P*>.05).

After adjusting for age and BMI, statistically significant reductions in RMSSD (*F*_2,10_=4.9757; *P*=.03) and LF relative power (*F*_2,10_=12.5032; *P*=.002) were observed in the infected group compared to both the healthy and post–COVID-19 condition groups, as confirmed through post hoc comparisons (Table 6). For SDNN (*F*_2,10_=2.4691; *P*=.13) and HF relative power (*F*_2,10_=0.8544; *P*=.45), no significant group differences were detected. Post hoc comparisons for these indexes showed marginal differences for SDNN (eg, post–COVID-19 condition vs infected; *P*=.01) but no significant differences for HF relative power (*P*>.05 in all cases). The lack of significance for SDNN compared to previous analyses in which it was significant (*P*=.01) may be attributable to the lower BMI values in this subset as BMI is known to influence HRV.

Age and BMI did not significantly impact most HRV indexes in the ANCOVA model, consistent with the nonsignificant effects observed for these covariates (*P*>.20). Homogeneity of variances (Levene test) and normality of residuals (Shapiro-Wilk test) were confirmed for all indexes, supporting the validity of the ANCOVA model.

The results indicate that participants with post–COVID-19 condition exhibited HRV patterns more similar to those of healthy individuals, whereas participants who were infected consistently showed lower RMSSD and LF relative power values. Although traditional statistical tests did not consistently distinguish participants with post–COVID-19 condition from healthy participants, the machine learning algorithms described in the Methods section uncovered nonlinear, multivariate HRV patterns, reinforcing their utility in detecting subtle autonomic dysregulation in post–COVID-19 conditions.

Classification models were initially implemented in MATLAB to categorize individuals into 3 groups—healthy, infected, and post–COVID-19 condition—using the dataset presented in Table 3. In this exploratory phase, all models were trained without restrictions on complexity, including the decision tree, SVM, k-NN, and neural network classifiers. This approach allowed for the identification of the most promising techniques for subsequent fine-tuning and refinement.

Hyperparameter optimization was then conducted using the *optimizer* function to enhance each model’s performance. Table 8 presents the overall classification accuracy before and after optimization using only the 4 physiological HRV indexes (SDNN, RMSSD, and LF and HF relative power).

Following this analysis, the decision tree model stood out as the most interpretable and promising candidate for further refinement. Table 9 shows detailed performance metrics for each class, revealing strong discriminative ability for the acutely infected group but limitations in distinguishing individuals with post–COVID-19 condition from healthy individuals.

To improve the model’s clinical realism and performance, a second training round was conducted using an additional variable, *covidrecent*. This binary contextual feature, coded as 1 only for participants with post–COVID-19 condition, mimics the temporal triage question routinely asked in practice (ie, recent infection within 3 months). Its inclusion significantly enhanced classification results.

At this point, the decision tree algorithm was reimplemented in Python to allow for full control over model complexity and ensure interpretability. Unlike the MATLAB phase, the Python implementation applied restrictions on model depth (*max_depth*) and number of features considered per node (*max_features*) to avoid overfitting and improve generalizability.

In total, 2 final models were retained: one trained only using the 4 HRV indexes (*max_depth*=3) and another trained using 5 inputs, including the *covidrecent* variable (*max_depth*=4). These hyperparameters were chosen based on cross-validation performance and the trade-off between accuracy and interpretability. Table 9 presents the performance metrics for the HRV-only model, and Table 10 presents the metrics for the improved model incorporating the *covidrecent* variable.

**Table 8:** Classification model results—before and after optimization.

Model	Accuracy before optimization (%)	Accuracy after optimization (%)
Decision tree	73.77	75.4
SVM^a^	73.77	73.77
KNN^b^	73.77	77
Neural networks	72.13	72.13

^a^SVM: support vector machine.

^b^KNN: k-nearest neighbor.

**Table 9:** Decision tree performance metrics by class (heart rate variability indexes only; macroaverage=76.4%).

Class	Precision (%)	Recall (%)	*F*_1_-score (%)	Area under the curve
Healthy	61	70	65	0.6841
COVID-19	86	90	88	0.8536
Post–COVID-19 condition	62	50	56	0.7543

**Table 10:** Classification model using Python—decision tree algorithm (macroaverage=87%).

Class	Precision (%)	Recall (%)	*F*_1_-score (%)	Area under the curve
Healthy	78	90	84	0.8695
Infected	89	81	85	0.8798
Post–COVID-19 condition	95	90	92	0.8421

Although the unrestricted MATLAB model showed slightly higher recall for participants who were infected, it also exhibited reduced sensitivity for post–COVID-19 condition. The Python-based models, although slightly less aggressive in fit, demonstrated better overall balance and clearer rule structures, an essential factor for clinical applications.

To evaluate the discriminatory potential of HRV indexes across clinical groups, a decision tree classifier was first implemented using scikit-learn. The input features consisted of the 4 HRV variables: RMSSD, SDNN, and LF and HF relative power. The target variable was the diagnostic class (0=control, 1=acute COVID-19, and 2=post–COVID-19 condition).

Model training and evaluation were conducted using 15-fold stratified cross-validation. The classifier used the Gini impurity criterion and a manually selected maximum depth of 3, which offered the best trade-off between accuracy and generalization. Table 9 summarizes the class-specific performance metrics, including precision, recall, *F*_1_-score, and AUC.

These results reflect the partial success of HRV-based classification. While the model captured robust physiological patterns for participants with acute infection, its ability to separate individuals with post–COVID-19 condition from healthy individuals remained limited when relying solely on HRV.

To address this, we incorporated a contextual temporal variable (*covidrecent*), indicating whether participants reported SARS-CoV-2 infection within the prior 3 months—a common clinical intake question—thereby reflecting a more realistic triage scenario.

Following this enhancement, the decision tree was reimplemented and re-evaluated in Python using five predictors (the four HRV indices plus *covidrecent*), resulting in improved balance and interpretability. Although other algorithms such as SVM showed slightly higher raw accuracy in previous MATLAB runs, decision tree was selected for its interpretability, an essential aspect in clinical decision-making contexts, in which transparency and traceability are critical [83,84].

The model’s performance was assessed using precision, recall, *F*_1_-score, and AUC, all derived from the confusion matrix. All values were expressed as percentages except for AUC, which was presented on a scale from 0 to 1. The final model achieved precision, recall, and *F*_1_-score values of 78%, 90%, and 84% for healthy participants; 89%, 81%, and 85% for participants who were infected; and 95%, 90%, and 92% for those with post–COVID-19 condition, respectively. The corresponding AUC scores were 0.8695, 0.8798, and 0.8421, respectively. The macroaverage of precision, recall, and *F*_1_-score was 87%, as shown in Table 10.

The Python-implemented decision tree model trained on the HRV patterns of 61 participants combined physiological HRV indexes (SDNN, RMSSD, and LF and HF relative power) with contextual information (*covidrecent*) to classify individuals into the healthy, infected, or post–COVID-19 condition groups with improved balance and interpretability.

To evaluate the discriminatory potential of HRV indexes across clinical groups, a decision tree classifier was implemented using scikit-learn. The input features consisted of 4 HRV variables: RMSSD, SDNN, and LF and HF relative power. The target variable was the diagnostic class (0=control, 1=acute COVID-19, and 2=post–COVID-19 condition).

**Figure 3:**
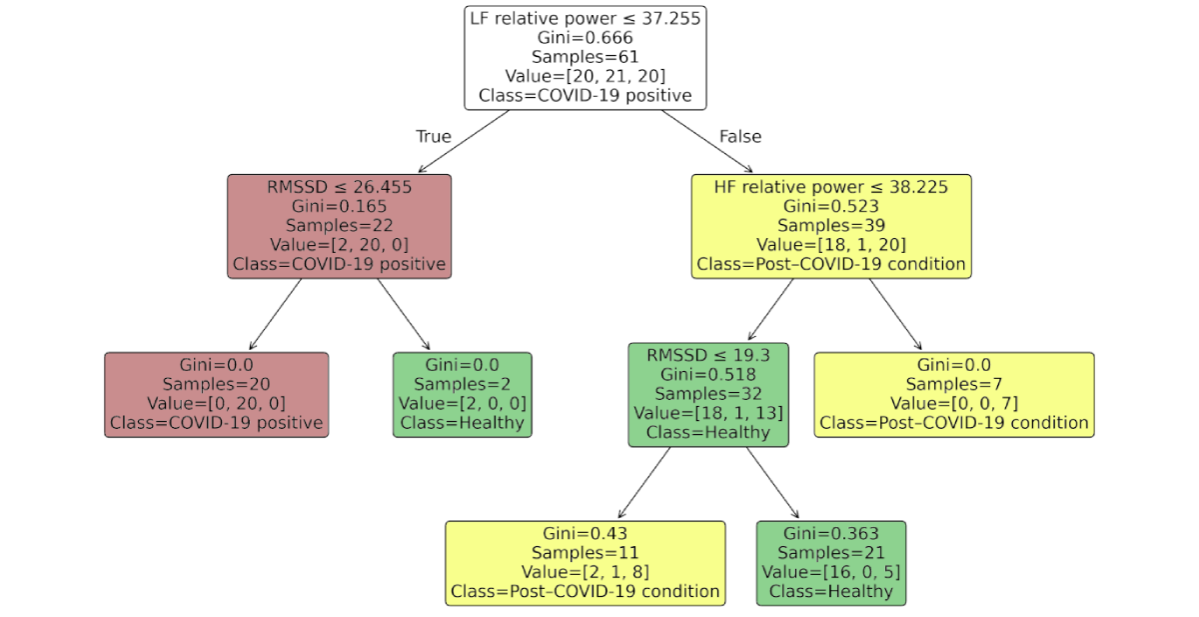
Decision tree classifier based on heart rate variability indexes alone (root mean square of successive differences [RMSSD], SD of the normal-to-normal intervals, low-frequency [LF] relative power, and high-frequency [HF] relative power) without the covidrecent variable.

Model training and evaluation were conducted using 15-fold stratified k-fold cross-validation to maximize robustness in performance estimation for this dataset. The classifier used the Gini impurity criterion and a maximum tree depth of 3 manually selected to balance model interpretability and generalization. Cross-validation predictions and performance metrics, including accuracy, are presented in Table 9. In addition to predictive performance, the model's structure was exported and visualized as a classification diagram (Figure 3), enabling intuitive interpretation of decision rules. Variable importance scores based on node impurity reductions are summarized in Table 11 to illustrate each HRV index’s relative contribution.

**Table 11:** Variable importance in the decision tree model trained without covidrecent.

Variable	Importance
LF^a^ relative power	0.58
RMSSD^b^	0.28
HF^c^ relative power	0.11
SDNN^d^	0.03

^a^LF: low frequency.

^b^RMSSD: root mean square of successive differences.

^c^HF: high frequency.

^d^SDNN: SD of the normal-to-normal intervals.

To evaluate the impact of adding contextual information, a second decision tree model was trained using the same HRV indexes plus the temporal variable *covidrecent* and a maximum tree depth of 4. This feature captured whether the participant had a confirmed SARS-CoV-2 infection within the previous 3 months, a piece of information commonly available at clinical intake and coded independently of the diagnostic label.

Inclusion of this feature improved the macroaverage classification accuracy from 76.4% to 87%, primarily by enhancing the model’s ability to distinguish post–COVID-19 condition cases from the control group. Importantly, this improvement did not result from data leakage or circular logic as *covidrecent* was derived from self-reported history, not the outcome variable.

**Figure 4:**
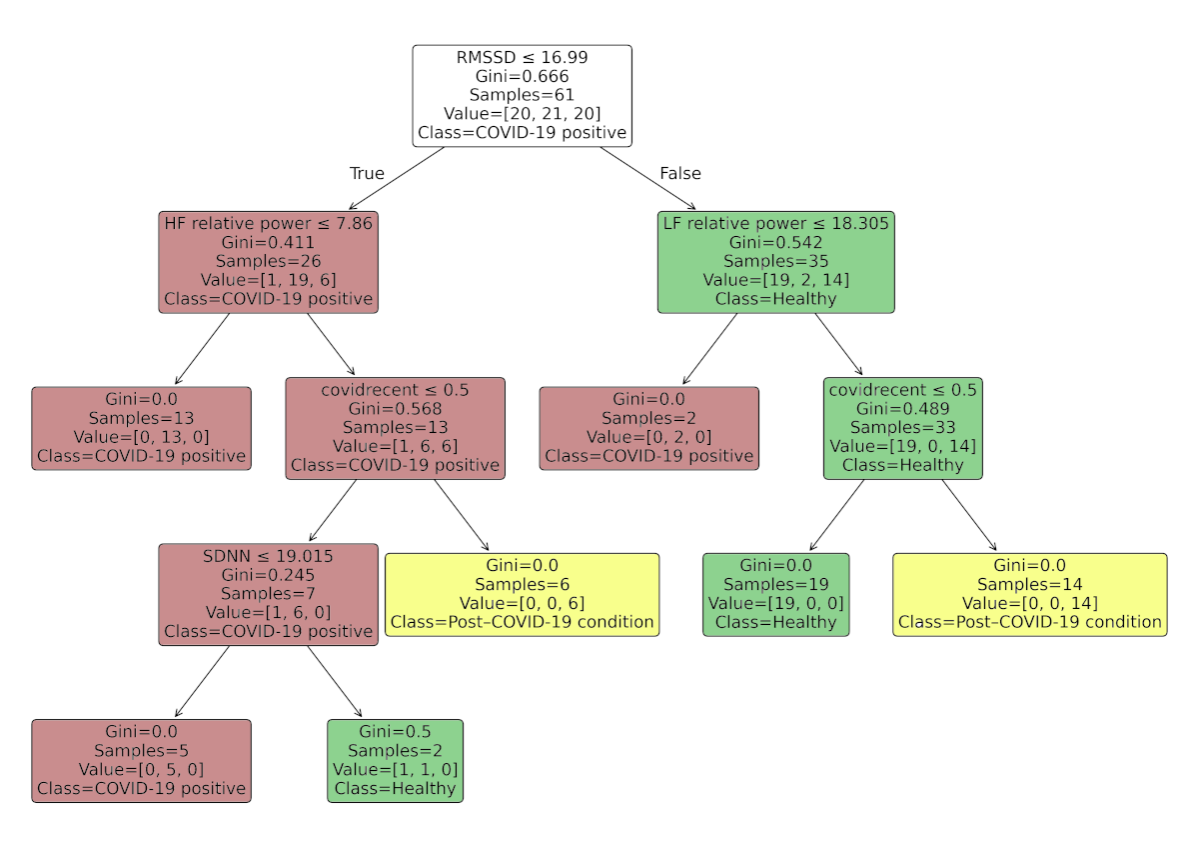
Decision tree classifier using both heart rate variability indexes and the covidrecent variable. HF: high frequency; LF: low frequency; RMSSD: root mean square of successive differences; SDNN: SD of the normal-to-normal intervals.

**Figure 5:**
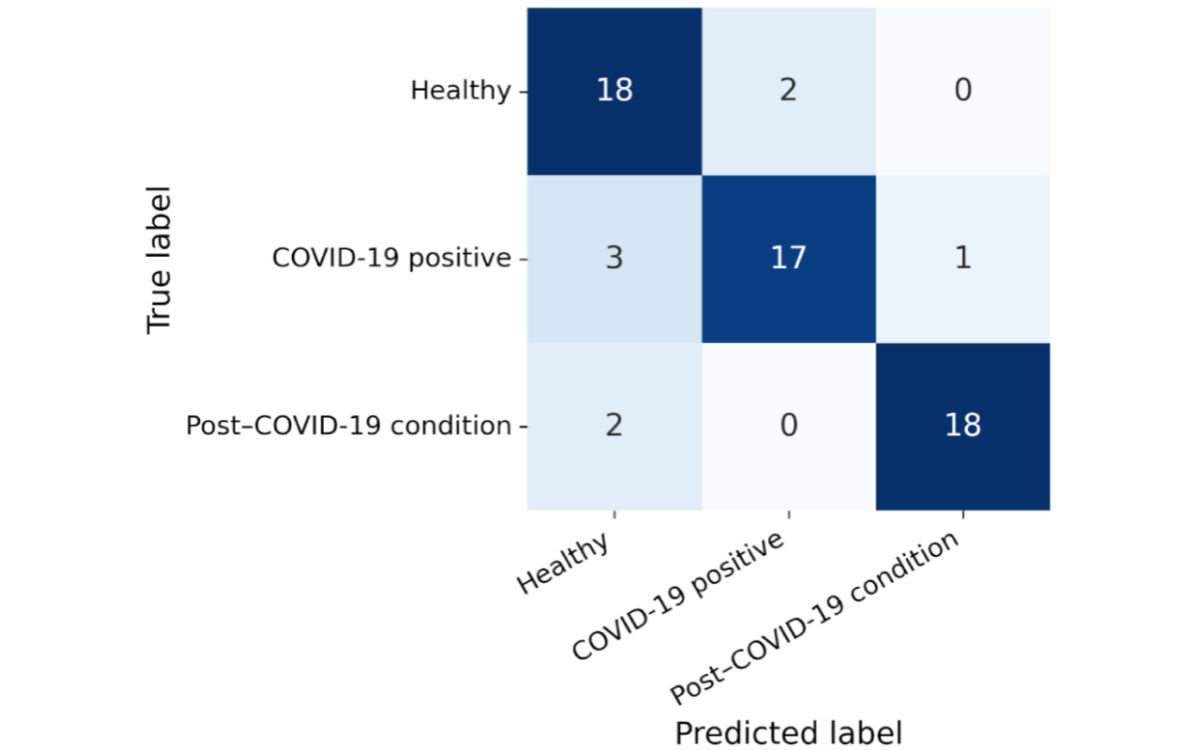
Confusion matrix for the decision tree model including the covidrecent variable. Class 0=healthy; class 1=infected; class 2=post–COVID-19 condition.

The updated classification diagram is shown in Figure 4, and the revised variable importance is summarized in Table 12, reflecting the integration of this variable. While *covidrecent* emerged as a dominant early-split feature, physiological HRV markers such as RMSSD and LF relative power retained significant roles. This suggests that combining objective and contextual inputs enhances triage potential without compromising biological plausibility. The corresponding confusion matrix is presented in Figure 5.

**Table 12:** Variable importance in the decision tree model including covidrecent.

Variable	Importance
“Covidrecent”	0.54
RMSSD^a^	0.28
HF^b^ relative power	0.09
LF^c^ relative power	0.08
SDNN^d^	0.02

^a^RMSSD: root mean square of successive differences.

^b^HF: high frequency.

^c^LF: low frequency.

^d^SDNN: SD of the normal-to-normal intervals.

When the *covidrecent* variable, indicating a confirmed SARS-CoV-2 infection within the previous 3 months, was unavailable, it was treated as null, reducing the model’s performance to the levels shown in Table 9. This highlights the role of the variable in enhancing discriminative power beyond core HRV patterns.

To further illustrate the effect of *covidrecent*, we generated visualizations of the decision rules, feature importance, and confusion matrices from the trained classifiers both with and without the additional variable.

Figure 3 shows the classification tree based solely on physiological indexes. The tree structure highlights the role of LF relative power and RMSSD in separating participants who were infected from healthy participants but fails to isolate post–COVID-19 condition cases effectively, reflecting the classification challenges observed when *covidrecent* was not included.

As shown in Table 11, LF relative power emerged as the most influential feature, followed by RMSSD and HF relative power. SDNN provided no meaningful contribution in this setting, likely due to its redundancy with RMSSD and HF power in short-term HRV measurements [70,85].

By including the *covidrecent* variable, the classification tree in Figure 4 shows a dramatic shift—this variable appears as the root node, immediately separating post–COVID-19 condition cases from others, simplifying subsequent splits based on LF relative power and RMSSD.

This behavior indicates that *covidrecent* had the highest discriminative value among the available features, guiding the model to prioritize this variable during decision tree construction. This is visually reinforced by the dominance of this variable in the feature importance rankings (Table 12).

As summarized in Table 12, the temporal variable *covidrecent* played a dominant role in the model’s decision structure. Nevertheless, physiological features such as RMSSD and HF and LF relative power continued to contribute meaningfully. Although SDNN appeared to have minimal importance, its presence reinforced the model’s ability to leverage multiple HRV dimensions even when some were partially redundant in short-term recordings.

The confusion matrix in Figure 5 illustrates the model’s improved classification accuracy. Most post–COVID-19 condition cases (18/20, 90%) were correctly identified, and only a few misclassifications occurred between healthy individuals and those who were infected. This reflects a substantial improvement compared to the model trained without the contextual variable.

These results reinforce the clinical utility of combining temporal and physiological inputs for triage decision-making. Precision-recall curves for all 1-vs–the rest binary classifications can be found in Multimedia Appendix 1, providing additional insights into performance across thresholds.

To illustrate the real-world applicability of the model, a near–real-time demonstration was conducted involving 4 participants: 2 (50%) healthy individuals and 2 (50%) hospitalized patients with COVID-19 with confirmed RT-PCR tests. All participants were recruited from the same medical unit as those in the training dataset. Their HRV indexes were collected following the established methodology and classified using the trained decision tree model.

This model, implemented in Python and trained on the HRV and contextual patterns of 61 participants, successfully classified all 4 test participants as either healthy or infected. No post–COVID-19 condition cases were available during this demonstration. The demographic characteristics of the participants are shown in Table 13, whereas Table 14 summarizes their HRV indexes.

**Table 13:** Demographic characteristics of near–real-time test participants.

Feature		COVID-19 (n=2)	Healthy (n=2)
**Sex, n (%)**			
	Female	1 (50)	1 (50)
	Male	1 (50)	1 (50)
**Age (y)**			
	Female participant	59	54
	Male participant	68	53
**Weight (kg)**			
	Female participant	45	60
	Male participant	65	90
**Height (cm)**			
	Female participant	152	166
	Male participant	160	183
**BMI (kg/m^2^)**			
	Female participant	19.5	21.8
	Male participant	25.4	26.9

**Table 14:** Heart rate variability indexes of near–real-time test participants.

Participant	Sex	Clinical condition	RMSSD^a^	SDNN^b^	LF^c^ relative power	HF^d^ relative power
P1	Female	Infected	18.14	24.54	20.2	30.5
P2	Male	Infected	19.43	24.57	38.33	39.41
P3	Female	Healthy	32.8	42.8	39	14.24
P4	Male	Healthy	33.13	33.12	29.16	12.47

^a^RMSSD: root mean square of successive differences.

^b^SDNN: SD of the normal-to-normal intervals.

^c^LF: low frequency.

^d^HF: high frequency.

Table 13 presents the demographic characteristics of the 4 participants in the near–real-time demonstration, including sex, age, weight, height, and BMI for the healthy and infected pairs. These factors allowed for comparison between the 2 groups, whereas Table 14 summarizes the HRV indexes (SDNN, RMSSD, and LF and HF relative power) collected during the test for each participant.

The near–real-time classification test, conducted using the trained decision tree model, correctly identified all 4 participants as either *infected* or *healthy*, achieving 100% accuracy for the available classes.

Trained on HRV patterns from 61 participants, the final model reached 87% overall accuracy in distinguishing among healthy individuals and those who were acutely infected and had post–COVID-19 condition. These results underscore the model’s practical utility and reinforce the potential of combining physiological and contextual data to support fast, noninvasive triage in clinical and remote monitoring scenarios.

## Discussion

### Principal Findings

This study demonstrates that HRV can be effectively used to distinguish among healthy individuals, those with active COVID-19, and those with post–COVID-19 condition, in alignment with previous findings on HRV reduction during and after infection. The observed differences in SDNN, RMSSD, and LF and HF relative power among these groups provide a robust basis for developing predictive models that advance beyond existing approaches [37-41,43-53,56-62].

The selection of HRV indexes varies across studies and influences their interpretation. Previous research has often relied on absolute LF and HF power [37,47,62,86], an approach known to depend on the individual’s basal heart rate, as highlighted by the Task Force of the European Society of Cardiology and the North American Society of Pacing and Electrophysiology [66] and Kamath et al [72], introducing variability when comparing populations. Normalized units such as LFnu and HFnu [74] face similar limitations. Other studies have used only time-domain indexes such as SDNN or RMSSD [46,50]. In contrast, our study examined SDNN, RMSSD, and LF and HF expressed as relative power (LF% and HF%), consistent with recommendations from [66,72,75] that suggest these metrics enhance comparability across diverse groups.

A time frame for physiological data acquisition is a key consideration in HRV analysis. Data can be collected in short- or long-term settings, each with distinct methodological implications. Short-term HRV studies are conducted under controlled conditions, where confounding factors such as body position, physical activity, respiration, and environmental temperature are monitored and minimized [70]. In contrast, long-term HRV data are typically acquired using wearable sensors (eg, Holter monitors), allowing participants to move freely during daily activities. These methodological differences significantly influence HRV indexes in both the time and frequency domains [66], and their physiological significance may differ as short-term HRV measures correlate only weakly with long-term values [72]. Such variation in measurement protocols can lead to discrepancies in findings across studies. Of the studies cited in this work that assessed HRV in COVID-19 contexts, approximately 59% used short-term protocols.

Aging is a well-established factor associated with the reduction in HRV. Several studies have documented that advancing age is linked to a progressive decline in specific HRV indexes, particularly RMSSD, SDNN, and the HF and LF components [68,78,79]. This reduction primarily reflects a decrease in parasympathetic activity, although global variability is also affected. However, the magnitude of HRV decline associated with aging occurs gradually and progressively, with modest annual changes [77] particularly after the fifth decade of life.

Beyond aging, pathological conditions such as SARS-CoV-2 infection have also been associated with significant reductions in HRV.

Time- and frequency-domain HRV indexes can be influenced by multiple factors, including age-related autonomic changes [87,88], which may complicate comparisons across groups with differing age distributions. Nevertheless, several studies have reported that both acute COVID-19 and post–COVID-19 condition can independently impact HRV [37,49,62,89], reinforcing its potential relevance for identifying autonomic dysfunction in these populations.

In this study, although the COVID-19 group exhibited a higher mean age and BMI compared to controls, the pronounced reductions in HRV indexes substantially exceeded the variations typically attributed to differences in age or BMI alone. Moreover, the BMI distribution within the COVID-19 group was relatively homogeneous without extreme outliers, minimizing the potential for confounding effects related to body composition. These findings suggest that acute SARS-CoV-2 infection likely represented the primary driver of the autonomic dysfunction observed in this cohort rather than demographic or anthropometric factors.

The ANCOVA with PSM confirmed that the reductions in RMSSD and LF relative power in the infected group were primarily driven by SARS-CoV-2 infection independent of age and BMI. By balancing age and BMI across groups, the analysis mitigated the confounding effects of these demographic factors. These findings align with those of previous studies reporting HRV reductions in acute COVID-19, suggesting autonomic dysfunction potentially due to inflammation or direct viral effects on the ANS [37,89].

Although SDNN is widely recognized as a reliable marker of autonomic dysfunction, especially in acute COVID-19, its importance in classifying features in our decision tree model was minimal. This likely reflects its high collinearity with RMSSD in short-duration HRV recordings [70,85,90]. This outcome does not diminish the physiological value of SDNN but rather illustrates how decision tree algorithms may underrepresent physiologically relevant variables when they are statistically correlated with others, particularly in short-term contexts.

Algorithm selection in our study balanced practical and methodological factors. Decision trees were chosen for their simplicity and interpretability. This choice aligns with approaches in prior work, such as the study by Vellido [84], which emphasizes interpretability in medical contexts, and contrasts with studies such as the one by Aliani et al [37] that may use more complex techniques to detect subtle autonomic changes. Meanwhile, the study by Teng et al [83] highlights how decision trees provide rule-based clarity, offering a lens to examine HRV patterns across COVID-19 states, although this approach reveals a trade-off between model simplicity and the broader applicability of findings when compared to other studies.

In summary, factors such as data collection duration, participant demographics, and the selection of HRV indexes and algorithms shape the interpretation of HRV findings and their comparability with those of prior work. Our study builds on existing research [37-42,55-62] by integrating short-term HRV measures and relative power indexes (LF and HF) to delineate COVID-19–related patterns in a cohort of 61 participants, yet it reveals methodological differences that complicate direct comparisons.

For instance, while the study by Aliani et al [37] used random forest and SVM to detect subtle autonomic shifts in COVID-19 severity using HRV data derived from PPG, and other studies [46,50,51] report autonomic dysfunction in post–COVID-19 condition through extended 24-hour Holter monitoring, our short-term approach captures acute differences among healthy, infected, and post–COVID-19 condition groups. These contrasts suggest that standardizing these factors could refine future investigations into HRV as a biomarker for COVID-19 and beyond.

Building on these foundations, this study advances the field through 2 key contributions. First, it delineates a potential physiological signature derived from specific HRV indexes capable of distinguishing healthy individuals; patients with active COVID-19; and those with post–COVID-19 condition, a condition currently diagnosed solely through clinical criteria due to the lack of objective biomarkers. Second, it introduces a fully automated, noninvasive classification system leveraging near–real-time HRV data to identify both acute COVID-19 and post–COVID-19 condition.

The integration of the temporal variable *covidrecent* into the classification framework provided an important insight for clinical triage and decision support. While traditional statistical analyses (eg, Mann-Whitney *U* tests) did not show significant differences in HRV indexes between individuals with post–COVID-19 condition and healthy individuals, the decision tree model identified multivariate patterns that separated these groups. The model achieved 90% recall and 92% *F*_1_-score for the post–COVID-19 condition class, demonstrating that combining contextual clinical information with physiological data can improve diagnostic accuracy.

Although HF relative power did not retain statistical significance after adjusting for age and BMI in the ANCOVA, it was selected by the decision tree model as an early-split feature. This apparent discrepancy reflects the fundamental difference between statistical inference and predictive modeling. Machine learning algorithms prioritize features based on their contribution to classification accuracy and reduction in node impurity rather than isolated group comparisons. In this context, HF relative power contributed to a decision threshold that helped differentiate post–COVID-19 condition from other classes even if its adjusted group means were not statistically distinct. This underscores the strength of multivariate pattern recognition in revealing complex physiological dynamics, particularly in heterogeneous syndromes such as post–COVID-19 condition.

These insights are especially relevant when considering the practical deployment of HRV-based classification systems. In demonstrating these contributions, this study also established the feasibility of an autonomous near–real-time HRV-based health monitoring system that is fully operational. The system’s performance depends primarily on the robustness and generalizability of the physiological signatures identified rather than on its technical architecture. Further validation with larger and more diverse populations would refine the HRV patterns used, enhancing the clinical applicability of the system.

### Limitations

This study is subject to several limitations that should be considered when interpreting its findings. The sample size of 61 participants (n=21, 34% with active COVID-19; n=20, 33% healthy individuals; and n=20, 33% with post–COVID-19 condition) may limit the generalizability of the identified HRV patterns to larger and more diverse populations. Although age, sex, and comorbidities were recorded, no subgroup analysis was conducted to evaluate their specific influence on HRV, limiting insights into potential effect modifiers.

The HRV indexes for healthy participants and those with post–COVID-19 condition were derived from preprocessed data, preventing verification of whether the original RR interval measurements were affected by artifacts such as body movements, improper finger oximeter placement, or environmental interference. Furthermore, HRV data for participants with active COVID-19 were collected at a single research center, which may introduce geographic or institutional bias and reduce the representativeness of the findings.

The reliance on short-term HRV recordings, which were collected under controlled conditions using a custom finger oximeter, differs from long-term monitoring approaches such as 24-hour Holter recordings in both methodology and physiological interpretation. While this short-term approach effectively captured distinct HRV patterns for immediate classification, the lack of longitudinal data restricts the ability to assess changes in autonomic function over time in COVID-19 or post–COVID-19 condition progression.

The near–real-time health monitoring system demonstrated technical feasibility by accurately classifying 4 independent participants (n=2, 50% healthy and n=2, 50% with active COVID-19) based on HRV signatures derived from the main study cohort (N=61). While this supports the system’s operability, the underlying physiological patterns have not yet been validated across larger and more diverse populations. Therefore, the limitation lies not in the system itself but in the need for broader validation of the HRV-based signatures used for classification.

The classification model’s strong reliance on the temporal variable *covidrecent*, as shown by the drop in accuracy from 87% to 76.4% when it was removed, suggests that HRV patterns may require complementary clinical context to support robust classification, particularly for distinguishing post–COVID-19 condition. This highlights the need for further refinement and validation of physiological signatures across different clinical scenarios.

Finally, the validation strategy used in this study relied on stratified 15-fold cross-validation within a combined dataset of 61 participants, a decision driven by the need to preserve class balance and optimize statistical power across the 3 diagnostic groups. While this approach improves reliability over simpler methods, it cannot entirely eliminate the risk of performance inflation due to possible information leakage across folds, particularly in modest samples. Future studies with larger and more diverse populations are needed to support external validation and further assess the generalizability of the proposed models.

### Conclusions

This study advances the understanding of HRV as a biomarker by identifying multivariate physiological signatures—combinations of SDNN, RMSSD, and LF and HF relative power—that differentiate healthy individuals, those with active COVID-19, and those with post–COVID-19 condition. Rather than focusing on isolated HRV reductions, this approach captured integrated patterns across time- and frequency-domain metrics.

Machine learning models trained solely on HRV indexes achieved 76.4% accuracy, with high discriminative power for active COVID-19 (*F*_1_-score of 88% and AUC of 0.8536). However, the detection of post–COVID-19 condition cases was limited, with an *F*_1_-score of 56%, reflecting the greater subtlety and variability in the autonomic changes associated with this condition. When the contextual variable *covidrecent*, indicating a confirmed SARS-CoV-2 infection in the previous 3 months, was incorporated, the model’s performance improved substantially. Overall accuracy increased to 87%, and the classification of post–COVID-19 condition cases improved markedly, with the *F*_1_-score increasing from 56% to 92% and the AUC increasing from 0.7543 to 0.8421 while preserving the model’s high ability to distinguish individuals with active COVID-19 from healthy individuals.

Finally, this study demonstrated the feasibility of applying a trained model to new short-term HRV data under near–real-time conditions. The ability to acquire, process, and classify signals on demand underscores the value of adaptive health monitoring in both clinical and remote environments. These physiological signatures derived from time- and frequency-domain HRV features help bridge the current gap in objective diagnostic frameworks for post–COVID-19 condition and contribute to early detection in acute COVID-19. With broader validation, they may enable scalable, data-driven solutions for individualized care, aligned with emerging trends in digital and predictive health care.

## Appendix

Multimedia Appendix 1 The full heart rate variability (HRV) input dataset used for model training and testing including the covidrecent variable; detailed hyperparameter configurations and optimization results for all classification models without covidrecent; CIs for effect sizes across HRV indexes based on the Fisher Z transformation; and precision-recall curves for each pairwise classification task, supporting the evaluation of model performance under class imbalance conditions.

Multimedia Appendix 2 Additional technical details regarding the hardware architecture, signal processing, and transmission protocols.

## Abbreviations

ANCOVA: analysis of covariance
ANS: autonomic nervous system
AUC: area under the curve
CNN: convolutional neural network
CRP: C-reactive protein
ECG: electrocardiography
HF: high frequency
HRV: heart rate variability
LF: low frequency
M-RSA: maneuver of accentuation of respiratory sinus arrhythmia
PPG: photoplethysmography
PSM: propensity score matching
RMSSD: root mean square of successive differences
RNN-LSTM: recurrent neural network–long short-term memory model
RT-PCR: reverse transcription polymerase chain reaction
SDNN: SD of the normal-to-normal intervals
SVM: support vector machine

## Data Availability

All data analyzed during this study are included in this published article and its Multimedia Appendix 1. The raw RR interval datasets generated during this study are not publicly available due to participant privacy but are available from the corresponding author upon reasonable request.
