# Supplementary material for: Classification of Individuals With COVID-19 and Post–COVID-19 Condition and Healthy Controls Using Heart Rate Variability: Machine Learning Study With a Near–Real-Time Monitoring Component
Source: J Med Internet Res. 2025 Aug 14;27:e76613. doi: 10.2196/76613 (PMC12395111; doi:10.2196/76613)

Multimedia Appendix 1: Machine Learning Model Optimization and HRV Input Data for COVID-19 Health Monitoring."

This appendix provides detailed results of machine learning model optimization and the HRV input dataset used to differentiate healthy, acutely infected, and long COVID participants, as described in the main manuscript.

Table A1 – Model optimization results without the Covidrecent variable, showing accuracy before and after hyperparameter tuning.

Table A2 – HRV input dataset used for classification, including RMSSD, SDNN, LF%, HF%, and Covidrecent for healthy (Group 0), acutely infected (Group 1), and long COVID (Group 2) participants.

Table A3 shows the 95% confidence intervals for the effect sizes, which were estimated using Fisher's Z transformation.

Table A1 – Model Optimization Summary

Model	Accuracy before optimization (%)	Accuracy after optimization (%)	Optimized Hyperparameters
Ensemble	75.41	77.0	Ensemble method: AdaBoost; Maximum number of splits: 3; Number of learners: 41; Learning rate: 0.2478
Decision Tree	73.77	75.4	Maximum number of splits: 5; Split criterion: Maximum deviance reduction
SVM	73.77	73.77 (no improvement)	Kernel function: Gaussian; Kernel scale: 2; Box constraint level: 1; Multiclass coding: One-vs-One; Standardize data: Yes
KNN	73.77	77.0	Number of neighbors: 31; Distance metric: Euclidean; Distance weight: Inverse; Standardize data: No
Neural Network	72.13	72.13 (no improvement)	Trilayered Neural Network; Number of fully connected layers: 3; Layer sizes: 10, 10, 10; Activation: ReLU; Iteration limit: 1000; Regularization strength (Lambda): 0; Standardize data: Yes
Kernel	70.49	70.49 (no improvement)	Preset: Logistic Regression Kernel; Learner: Logistic Regression; Number of expansion dimensions: Auto; Regularization strength (Lambda): Auto; Kernel scale: Auto; Multiclass coding: One-vs-One; Standardize data: Yes; Iteration limit: 1000

Table A2, show data from 61 participants: 20 healthy, 21 acutely infected, 20 long COVID

Table A2 - HRV Input Data

Group	RMSSD (ms)	SDNN (ms)	LF (%)	HF (%)	Covidrecent
0	25.6	33.4	73.0	21.79	0
0	21.2	30.2	71.39	26.15	0
0	25.5	33.4	75.73	19.28	0
0	31.2	22.1	49.4	37.61	0
0	52.8	55.7	54.3	36.97	0
0	36.1	44.1	60.74	37.01	0
0	24.8	33.7	77.03	17.45	0
0	26.4	36.6	57.41	34.97	0
0	34.8	37.4	20.27	68.62	0
0	8.8	19.7	62.58	14.27	0
0	20.8	26.1	76.54	21.71	0
0	24.0	26.3	60.82	37.81	0
0	21.6	39.4	68.19	10.21	0
0	28.4	41.3	64.22	23.93	0
0	20.7	24.6	67.81	25.98	0
0	17.6	22.4	76.58	19.93	0
0	30.0	45.5	50.74	35.89	0
0	47.8	53.4	68.71	30.0	0
0	32.6	41.0	59.23	35.05	0
0	29.4	26.9	35.38	49.68	0
1	9.74	16.07	23.6	6.78	0
1	9.72	23.47	19.07	3.17	0
1	13.95	21.11	23.87	39.01	0
1	10.65	15.45	43.77	8.89	0
1	13.17	18.33	16.68	26.78	0
1	16.38	23.96	19.35	4.59	0
1	3.88	9.26	13.65	3.77	0
1	14.06	25.75	11.58	5.5	0
1	10.83	19.94	11.62	5.59	0
1	13.56	18.07	30.85	24.12	0
1	20.88	26.25	6.59	50.7	0
1	3.07	10.94	15.12	1.58	0
1	8.28	10.96	23.99	13.34	0
1	23.51	18.22	16.34	56.16	0
1	5.45	9.67	10.37	7.55	0
1	8.98	46.34	8.58	0.78	0
1	2.37	2.06	7.76	13.23	0

1	10.45	13.89	7.47	4.41	0
1	4.48	10.49	11.42	1.09	0
1	7.03	30.86	7.3	2.27	0
1	7.78	15.85	36.1	5.19	0
2	10.7	18.4	89.28	8.34	1
2	24.7	32.4	76.69	22.03	1
2	17.8	27.9	71.85	21.2	1
2	34.1	43.1	55.36	37.53	1
2	21.2	28.1	38.41	56.17	1
2	16.2	22.8	61.91	30.97	1
2	6.9	15.6	58.98	9.61	1
2	13.8	19.6	80.12	18.51	1
2	17.9	26.9	49.07	29.25	1
2	7.2	15.1	85.15	8.17	1
2	9.7	16.7	66.71	16.6	1
2	34.6	30.9	49.51	48.29	1
2	30.6	49.6	70.27	20.98	1
2	27.6	33.8	50.12	45.94	1
2	31.0	38.3	58.35	38.64	1
2	28.6	37.0	66.35	19.57	1
2	26.7	27.8	41.67	19.92	1
2	28.4	35.0	51.04	43.15	1
2	24.2	36.0	52.3	40.74	1
2	27.3	27.6	44.69	49.08	1

Note:
The column “Group” represents the target classes used in classification: 0 = healthy, 1 = acutely infected, 2 = long COVID. The variable “Covidrecent” was coded as 1 only for long COVID participants (Group = 2), and 0 for both other groups, either due to the absence of prior infection or uncertainty regarding timing.

Table A3 - Confidence intervals for the effect sizes were estimated using Fisher's Z transformation.

HRV Index	Group Comparison	Effect Size (r)	95% CI Lower	95% CI Upper
SDNN	Healthy vs COVID	0.68	0.471	0.817
SDNN	Healthy vs Long COVID	0.233	-0.085	0.508
SDNN	COVID vs Long COVID	0.538	0.276	0.726
RMSSD	Healthy vs COVID	0.77	0.606	0.871
RMSSD	Healthy vs Long COVID	0.244	-0.073	0.516
RMSSD	COVID vs Long COVID	0.595	0.352	0.763
LF%	Healthy vs COVID	0.823	0.69	0.902
LF%	Healthy vs Long COVID	0.094	-0.224	0.394
LF%	COVID vs Long COVID	0.847	0.729	0.916
HF%	Healthy vs COVID	0.546	0.286	0.731
HF%	Healthy vs Long COVID	0.026	-0.288	0.335
HF%	COVID vs Long COVID	0.53	0.266	0.72

To complement the evaluation metrics reported in the main manuscript, we present precision-recall (PR) curves for the three pairwise binary classifications using only HRV indices (i.e., without the contextual variable 'covidrecent'):

- Figure A1: Healthy vs Acute – Average Precision (AP) = 0.81
- Figure A2: Healthy vs Long COVID – AP = 0.58
- Figure A3: Acute vs Long COVID – AP = 0.83

These plots provide a threshold-independent visualization of model performance, especially useful in scenarios with class imbalance.

Figure A1. Precision-Recall Curve – Healthy vs Acute (AP = 0.81)

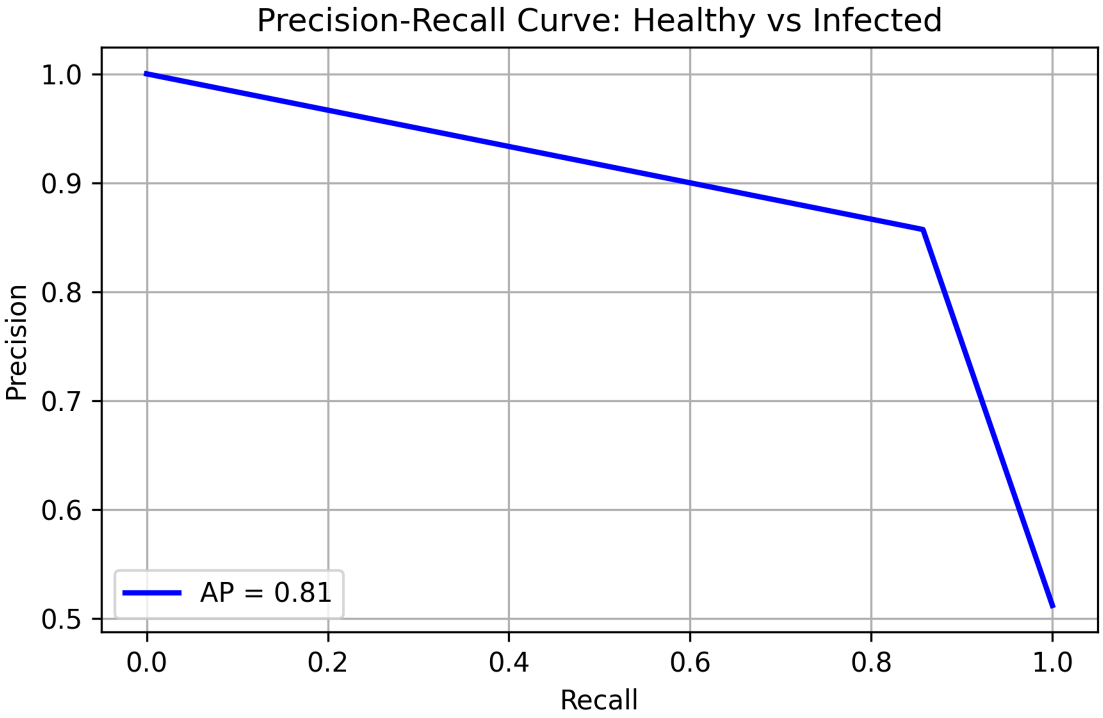

Figure A2. Precision-Recall Curve – Healthy vs Long COVID - (AP = 0.55)

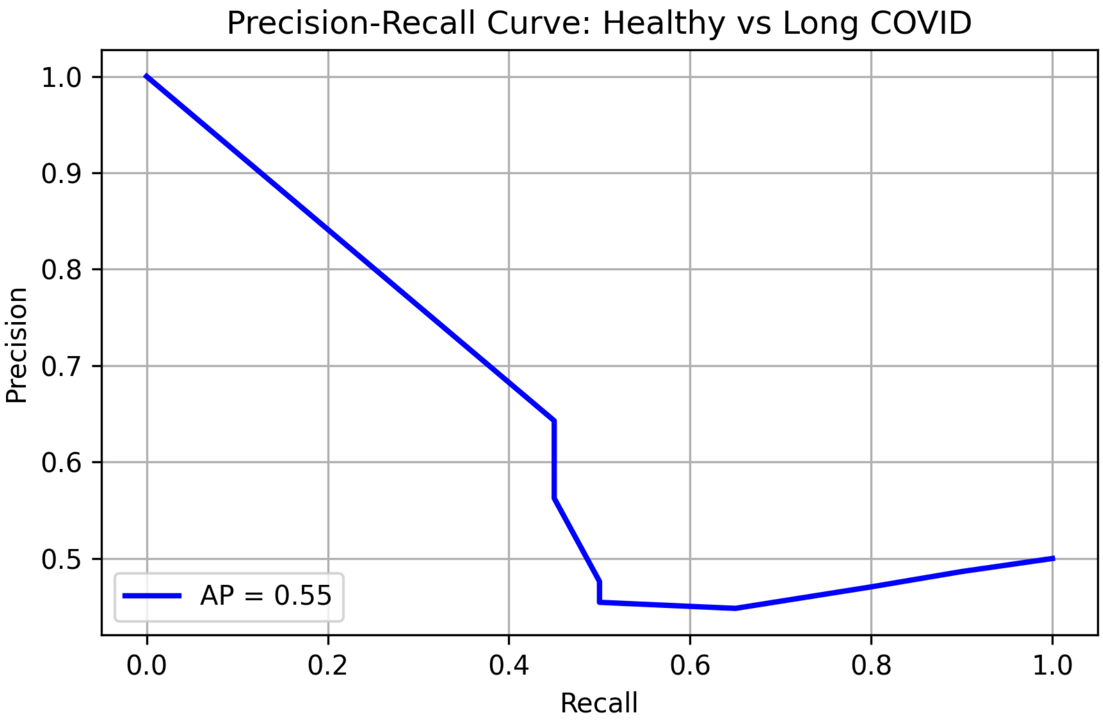

Figure A3. Precision-Recall Curve – Acute vs Long COVID - (AP = 0.90)

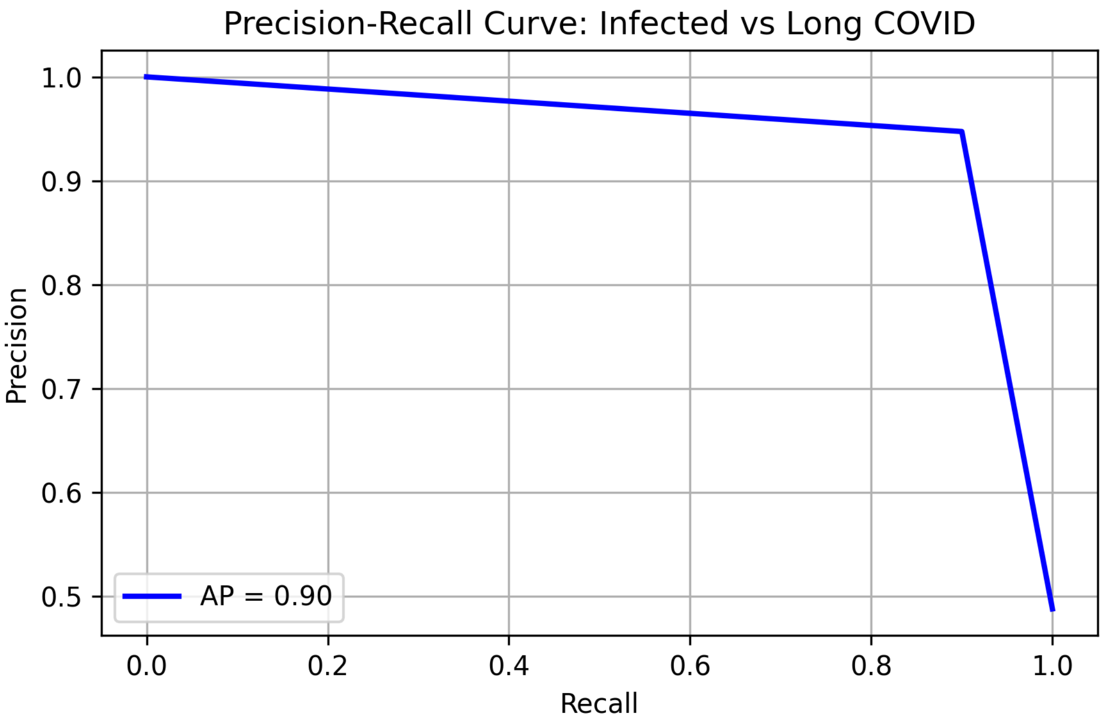

Supplement: Multimedia Appendix 1 [file jmir_v27i1e76613_app1.pdf]
