# Supplementary material for: Classification of Individuals With COVID-19 and Post–COVID-19 Condition and Healthy Controls Using Heart Rate Variability: Machine Learning Study With a Near–Real-Time Monitoring Component
Source: J Med Internet Res. 2025 Aug 14;27:e76613. doi: 10.2196/76613 (PMC12395111; doi:10.2196/76613)
Supplement: Multimedia Appendix 2 [file jmir_v27i1e76613_app2.pdf]

Multimedia Appendix 2: Technical Specifications of Custom Oximeter System for Near-Real-Time Health Monitoring

This appendix provides a comprehensive technical description of the custom-designed oximeter system introduced in the main manuscript, focusing on hardware architecture, signal processing, data transmission, and preliminary testing. While some contextual details are reiterated for clarity, this document emphasizes implementation specifics and analytical methods not covered in the primary text.

To enable near-real-time physiological monitoring, a bespoke oximeter was developed to capture photoplethysmography (PPG) signals and transmit raw interbeat (RR) interval data via Wi-Fi to a MySQL database. Unlike commercial oximeters, which typically provide only blood oxygen saturation (SpO_2) and beats per minute (BPM) without raw RR intervals, this system supports heart rate variability (HRV) analysis by delivering unprocessed PPG data. Specialized devices like the Polar V800 or Elite HRV offer RR interval measurements but require manual data transfer, limiting their suitability for automated, continuous monitoring. The custom system overcomes these constraints by enabling minute-by-minute data transmission using the MQTT protocol.

Hardware and Data Transmission

The oximeter hardware records PPG signals at 25Hz sampling rate, concatenating data into two-minute segments to ensure reliable low-frequency (LF) HRV component analysis, as recommended in literature [1] . Data are transmitted wirelessly via MQTT, a lightweight messaging protocol optimized for real-time applications. Upon completion of each recording session, measurements are automatically uploaded to a MySQL database, enabling integration with downstream processing pipelines without user intervention.

Signal Preprocessing

Raw PPG signals exhibit variability in amplitude across individuals, necessitating normalization to facilitate inter-subject comparisons. The min-max normalization method is applied, defined as:

$$\bar{x_i} = \frac{x_i(t) - \min(x)}{\max(x) - \min(x)} \qquad (1)$$

Where x_i represents the original raw PPG signal, and $\bar{x_i}$ represents the normalized PPG signal scaled to a [0, 1] range. While effective in preserving amplitude relationships, this method is sensitive to outliers. To address this, the HeartPy library [2] implements peak detection with adaptive thresholding and outlier rejection, filtering erroneous RR intervals caused by motion artifacts or sensor noise.

Post-normalization, a second-order Butterworth band-pass filter (0.5–3.5 Hz) is applied to attenuate low-frequency baseline wander (e.g., from respiration) and high-frequency noise (e.g., from electrical interference). The filter's frequency response is given by:

$$H(f) = \frac{1}{\sqrt{1 + \left(\frac{f}{f_c}\right)^{2n}}} \qquad (2)$$

Where f is the frequency, f_c is the cutoff frequency, and n is the filter order. (set to 2 for computational efficiency). This configuration ensures minimal distortion of the PPG signal while maintaining a flat response within the passband.

HRV Analysis

The HeartPy library processes the normalized and filtered PPG signal to detect systolic peaks, which are used to compute RR intervals. Peak detection algorithms identify and exclude erroneous RR intervals caused by motion artifacts or sensor noise, enhancing the reliability of the processed signal. The resulting RR intervals are analyzed to extract HRV metrics in both time and frequency domains, including:

SDNN: Standard deviation of NN intervals.
RMSSD: Root mean square of successive differences.
LF%: Percentage of low-frequency. (Relative power)
HF%: Percentage of high-frequency. (Relative power)

These metrics are stored in the MySQL database and fed into a machine learning model (detailed in the main manuscript and Multimedia Appendix 1) for classification of physiological states (e.g., healthy, COVID-19, post-COVID).

System Workflow and Visualization

The system's workflow, illustrated in Figure 1, encompasses PPG signal acquisition, preprocessing, HRV extraction, machine learning classification, and result visualization via a web interface. This interface provides near-instantaneous feedback on physiological status, enabling near-real-time health monitoring.

Figure 1. Near-Real-Time Health Monitoring System Workflow

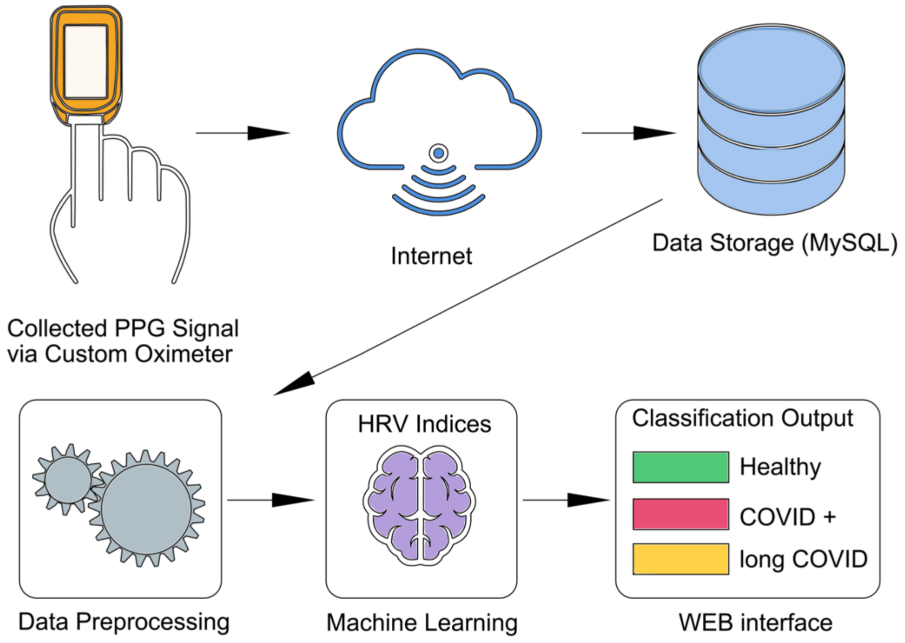

Preliminary Testing

To evaluate near-real-time functionality, the system was tested with four participants: two healthy individuals and two with RT-PCR-confirmed COVID-19, hospitalized in the same medical unit as the training dataset. Conducted in October 2024, this test coincided with a decline in severe COVID-19 cases, limiting participant numbers. Each two-minute recording session was followed by preprocessing, HRV computation, and classification, completed in approximately one second. Results, displayed via the web interface, confirmed the system's ability to replicate HRV-based classification patterns observed in the experimental dataset. However, given the small sample size, these findings represent a feasibility demonstration rather than a comprehensive validation.

Conclusion

The custom oximeter system enables near-real-time HRV-based health monitoring by integrating automated data acquisition, signal processing, and machine learning classification. Technical details provided here complement the main manuscript, offering insights into the system's design and implementation. Future work should focus on validating the system's performance across larger, more diverse populations to enhance generalizability.

References

1. Shaffer F, McCraty R, Zerr CL. A healthy heart is not a metronome: an integrative review of the heart's anatomy and heart rate variability. Front Psychol 2014 Sep 30;5. doi: 10.3389/fpsyg.2014.01040
2. van Gent P, Farah H, van Nes N, van Arem B. HeartPy: A novel heart rate algorithm for the analysis of noisy signals. Transp Res Part F Traffic Psychol Behav 2019 Oct;66:368–378. doi: 10.1016/j.trf.2019.09.015
